# Direct Observation of Phase Change Accommodating Hydrogen Uptake in Bimetallic Nanoparticles

**DOI:** 10.1021/acsnano.4c18013

**Published:** 2025-03-05

**Authors:** Lívia P. Matte, Maximilian Jaugstetter, Alisson S. Thill, Tara P. Mishra, Carlos Escudero, Giuseppina Conti, Fernanda Poletto, Slavomir Nemsak, Fabiano Bernardi

**Affiliations:** † Programa de Pós-Graduação em Física, Instituto de Física, Universidade Federal do Rio Grande do Sul (UFRGS), Porto Alegre 91501-970, Brazil; ‡ Advanced Light Source, Lawrence Berkeley National Laboratory, Berkeley, California 94720, United States; § Chemical Sciences Division, Lawrence Berkeley National Laboratory, Berkeley, California 94720, United States; ∥ Materials Science Division, Lawrence Berkeley National Laboratory, Berkeley, California 94720, United States; ⊥ ALBA Synchrotron Light Source, Cerdanyola del Vallès, Barcelona 08290, Spain; # Department of Physics and Astronomy, University of California, Davis, California 95616, United States; ∇ Departamento de Química Orgânica, Instituto de Química, Universidade Federal do Rio Grande do Sul (UFRGS), Porto Alegre 91501-970, Brazil

**Keywords:** hydrogen storage, morphology changes, bimetallic nanoparticles, in situ measurements, core−shell structure

## Abstract

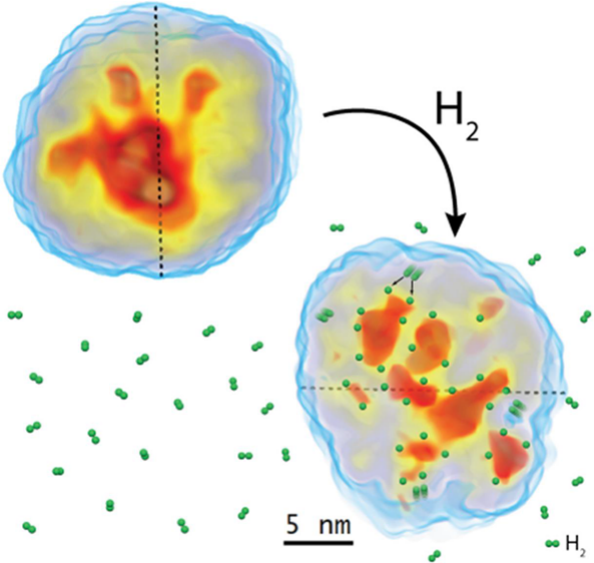

Hydrogen holds great promise as a cleaner alternative to fossil fuels, but its efficient and affordable storage remains a significant challenge. Bimetallic systems, such as Pd and Ni, present a promising option for storing hydrogen. In this study, using the combination of different cutting-edge X-ray and electron techniques, we observed the transformations of Pd–Ni nanoparticles, which initially consist of a NiO-rich shell surrounding a Pd-rich core but undergo a major transformation when they interact with hydrogen. During hydrogen exposure, the Pd core breaks into smaller pockets, dramatically increasing its surface area and enhancing the hydrogen storage capacity, especially in nanoparticles with lower Pd content. The findings provide a deep understanding of the morphological changes at the atomic level during hydrogen storage and contribute to designing cost-effective hydrogen storage using multimetallic systems.

The use of nonrenewable energy sources is directly related to climate change due to the emission of greenhouse gases. It is estimated that natural gas and oil reserves will be depleted before 2070,^1^ creating energy insecurity in economies based on these fuels. Thus, it is urgent to dramatically change the world’s energy matrix to a more renewable and sustainable one. Hydrogen is one of the most promising candidates to replace fossil fuels since it is available from renewable energy sources.^2^ It presents a gravimetric density 3 times bigger than gasoline, for example.^2^ Another benefit is that byproducts of energy generation using hydrogen consist mainly of H_2_O. However, the main challenge for the large-scale use of hydrogen as an energy carrier in light-duty vehicles is the hydrogen storage process. The currently available methods make it commercially disadvantageous. Hence, the US Department of Energy (DOE) set audacious targets for hydrogen storage materials that should be fulfilled by 2025, but we are still far away from reaching them.^3^

A promising way to store hydrogen is using solid materials,^4^ where the hydrogen can be either adsorbed at the surface or stored with the formation of a hydride phase. For instance, hydrogen can be adsorbed on the surface of activated carbon, which has a high gravimetric capacity but presents a very low volumetric capacity.^4^ On the other hand, complex metal hydrides, such as LiAlH_4_, present a high gravimetric and volumetric capacity, but too high desorption temperatures.^4^ Thus, the discovery of improved storage materials is still needed before full-scale commercialization can be realized.

The material used to adsorb hydrogen should present adsorption energy in the quasi-molecular bonding regime, between roughly 0.2 eV (19.3 kJ/mol H_2_) and 0.6 eV (57.9 kJ/mol H_2_).^5^ The major candidates are metallic nanostructures, where the adsorption energy can be adjusted by their morphology.^2^ Pd is known to have a high affinity with hydrogen and to form a stable Pd–H bond.^6^ However, the current Pd nanostructures do not get even close to fulfilling the DOE targets.^6^ Nevertheless, the adsorption energy may be adjusted also through the stoichiometry in bimetallic nanoparticles.^4^ Switching from monometallic to bimetallic nanostructures allows the creation of new atomic sites for hydrogen adsorption, thus improving the hydrogen storage capacity.^7^ Previously, we demonstrated that NiO nanofoams present a quasi-molecular bonding with hydrogen thereby presenting a lower hydrogen affinity compared to Pd.^8^ Therefore, a mixture of NiO and Pd in the form of Pd-NiO nanoparticles shows great promise for hydrogen storage applications. The inclusion of NiO is helpful also in reducing the cost compared with monometallic Pd. Previous studies demonstrated that the bimetallic Pd–Ni nanoparticles are great candidates for promoting efficient hydrogen storage.^9−11^ Thus, it is fundamental to thoroughly understand the atomic events occurring during hydrogen storage in these bimetallic nanoparticles to design improved hydrogen storage systems.

We herein present an approach utilizing complementary in situ techniques to determine atomic- and nanoscale transformations during the hydrogen storage process in Pd_*x*_Ni_100–*x*_ (*x* = 90, 75, 50, and 25) nanoparticles. Each technique is dedicated to probing different chemical and structural properties of the nanoparticles during the process. X-ray absorption spectroscopy (XAS) was used to observe the changes in the local atomic order around Ni and Pd atoms; X-ray photoelectron spectroscopy (XPS) probes the chemistry of components, and grazing incident X-ray scattering (GIXS) observed structural transformations. Measurements are complemented by electron energy loss spectroscopy (EELS) for nanoscale insights into the particles’ morphology and chemistry. The combination of all techniques reveals complex atomic mechanisms promoting hydrogen storage in these bimetallic nanoparticles. Furthermore, our results present a new cost-effective solution for hydrogen storage using Pd_*x*_Ni_100–*x*_ nanoparticles.

## Results and Discussion

**Figure 1:**
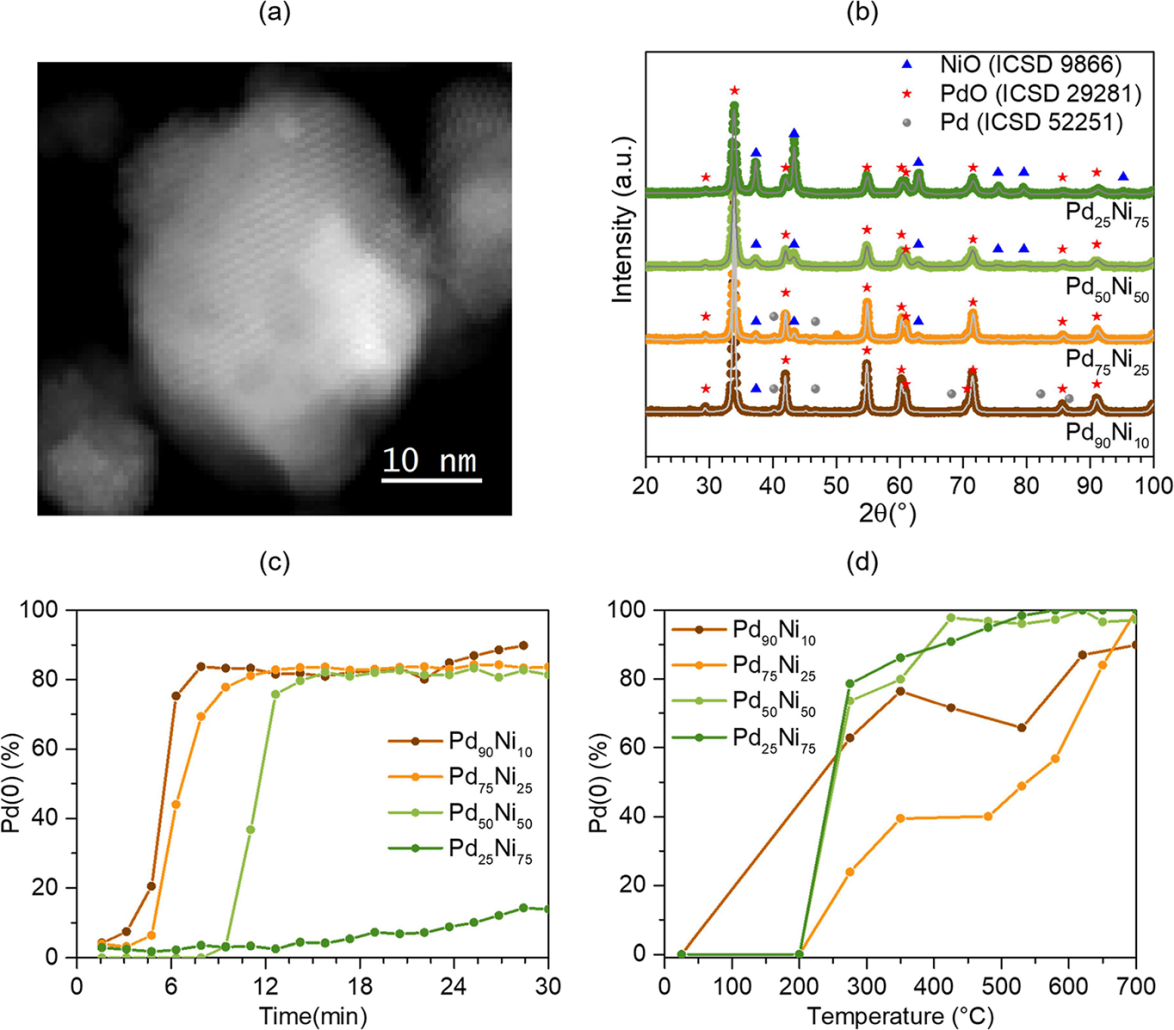
(a) Typical HAADF-STEM image of the Pd_25_Ni_75_ sample. (b) XRD measurements of the Pd_*x*_Ni_100–*x*_ samples. The points represent the experimental data, and the gray line represents the fit obtained from the Rietveld refinement. The blue triangle, red star, and gray circle show the position of the Bragg reflections related to the NiO, PdO, and Pd(0) crystalline phases, respectively. (c) Pd(0) fraction as a function of time obtained from in situ XANES measurements during the 30 mL/min 4% H_2_ + 96% He exposure at room temperature and (d) on the nanoparticles’ surface obtained from the XPS measurements under UHV as a function of temperature.

We performed ex situ characterization of morphology and composition of nanoparticles using high-angle annular dark field scanning transmission electron microscopy (HAADF-STEM) imaging. Figure 1a presents a typical image obtained for Pd_25_Ni_75_ nanoparticles, showing a nanoparticle with a diameter of around 20 nm, which is the mean value obtained for all samples from the TEM analysis, as presented in the histogram of the size distribution (Figure S1). Figure 1b presents the XRD measurements, indexing with the crystal phases, and Rietveld refinement. NiO and PdO phases are present in all samples, besides the presence of metallic Pd (Pd(0)) phase in smaller amounts in the Pd-richer samples, which agrees with XANES measurements in Figure S2. In general, NiO and PdO phases have similar crystallite sizes for all the samples (see Table S1). The XRD and TEM results show that the bimetallic nanoparticles exhibit similar diameter, morphology, and oxidation state between the samples. Moreover, the Pd content obtained from surface-sensitive XPS measurements in the as-prepared state is always smaller than that obtained from EDS measurements (Figure S3), indicating that all as-prepared samples present a core–shell-like structure with a Ni-rich shell and a Pd-rich core region. In addition, EDS maps show that Ni and Pd are well dispersed in the samples (Figure S4).

The reduction of Pd atoms is an essential step in the hydrogen storage process since Pd(0) has a much higher hydrogen storage capacity than PdO.^12^ The opposite occurs with Ni atoms, where NiO presents adsorption energy in the quasi-molecular bonding regime, while metallic Ni (Ni(0)) presents adsorption energy in the chemisorption regime.^8^Figure 1c shows the Pd(0) content obtained from the in situ XANES analysis as a function of time during the hydrogen exposure at RT (Figure S5). In line with previous reports, the initial high amount of PdO quickly reduces to Pd(0).^13,14^ Nevertheless, Pd is not fully reduced at the end of the process, in accordance with the literature that shows that the O diffusion process is too slow to fully reduce PdO to metallic Pd at room temperature.^15^ However, the time needed to reduce nanoparticles from PdO to Pd(0) strongly depends on the Pd/Ni ratio. The higher the amount of Pd, the faster the Pd reduction at RT under H_2_ exposure, suggesting that Ni plays an inhibitory role in the reduction process of Pd.

The near-surface region of the as-prepared samples exhibits only NiO and PdO chemical components, as observed by XPS (Figure S7). Figure 1d shows the atomic content of reduced Pd(0) as a function of temperature, obtained from the analysis of the AP-XPS spectra (Figure S8) measured under ultrahigh vacuum (UHV). It is observed that the total surface reduction occurs at higher temperatures for samples containing higher amounts of Pd. This is in contrast to what is observed under a H_2_ atmosphere.

**Figure 2:**
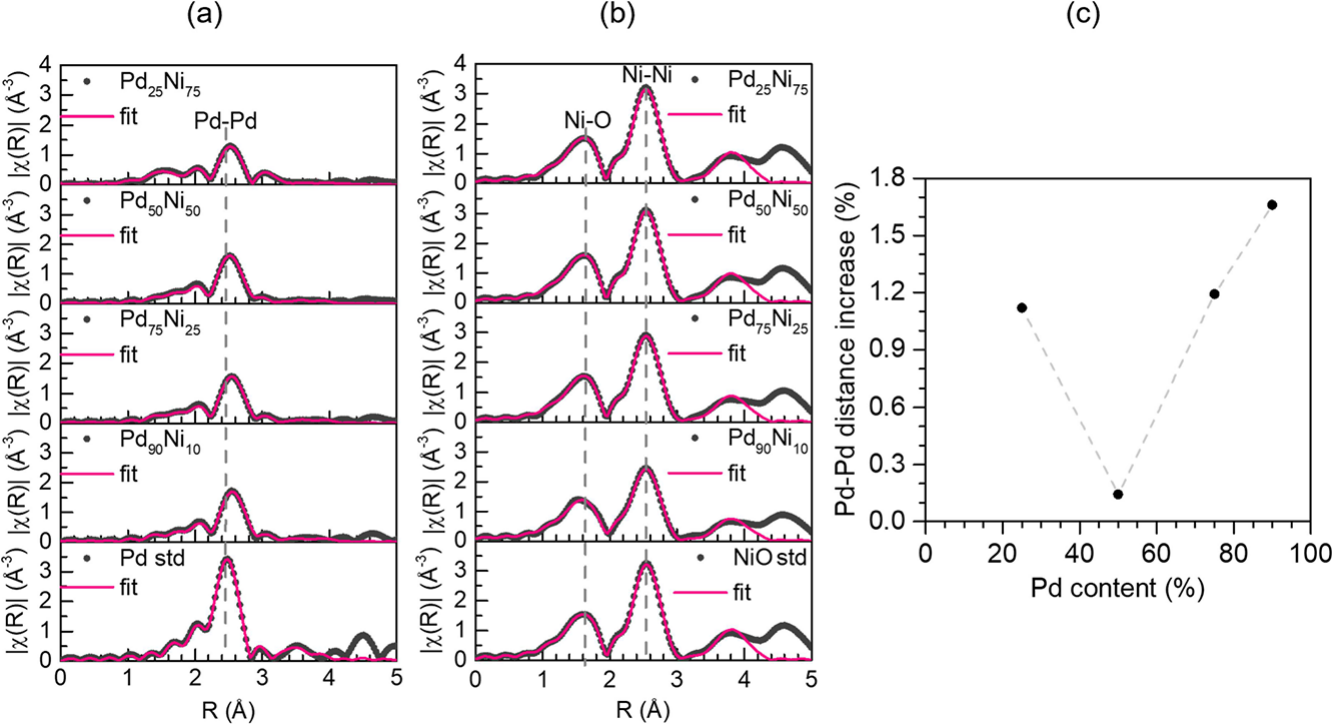
Fourier Transform of the EXAFS oscillations at the (a) Pd K edge and (b) Ni K edge during H_2_ exposure at RT and atmospheric pressure. The black dots represent the data measured, and the pink line represents the fit performed. (c) Increase of the Pd–Pd atomic distance in comparison to Pd standard position as a function of Pd content in the nanoparticles.

Aiming to observe the changes in the local atomic order around Ni and Pd atoms during the hydrogen storage process, we performed in situ EXAFS measurements. Figure 2 shows the Fourier Transform (FT) of the EXAFS oscillations (shown in Figures S9 and S10) for nanoparticles with different Pd/Ni ratios during H_2_ exposure at RT and atmospheric pressure. The FT data of all the samples are very similar to Pd(0) and NiO standards. However, the Pd_25_Ni_75_ sample also shows a small contribution from the Pd–O scattering path along with the Pd(0). Surprisingly, there is a clear decrease in the intensity of the peak related to the Pd–Pd scattering path in the nanoparticles in comparison to the Pd(0) standard, but this reduction in intensity does not occur for Ni–O and Ni–Ni paths. This is related to a reduced size or higher temperature of the sample. Since all measurements were conducted at RT, it shows some atomic rearrangement around Pd atoms different than Ni atoms. Under H_2_ exposure, the Pd–Pd scattering peak shifts to higher *R* values as compared to the Pd(0) standard, indicating lattice expansion upon H_2_ uptake. Furthermore, no shifts in the Ni–Ni or Ni–O scattering paths are observed compared to the NiO standard during H_2_ exposure.

Figure 2c displays the relative increase in the Pd–Pd scattering path distance from the coordination shell when compared to the Pd(0) reference (see Table S2 for the fitting parameters). There is an increasing trend of the Pd–Pd interatomic distance (up to a 1.7% increase) with the Pd amount, except for Pd_50_Ni_50_ nanoparticles that present only a slight increase. However, no shifts are observed in the Ni–Ni or Ni–O scattering peaks (Table S3).

**Figure 3:**
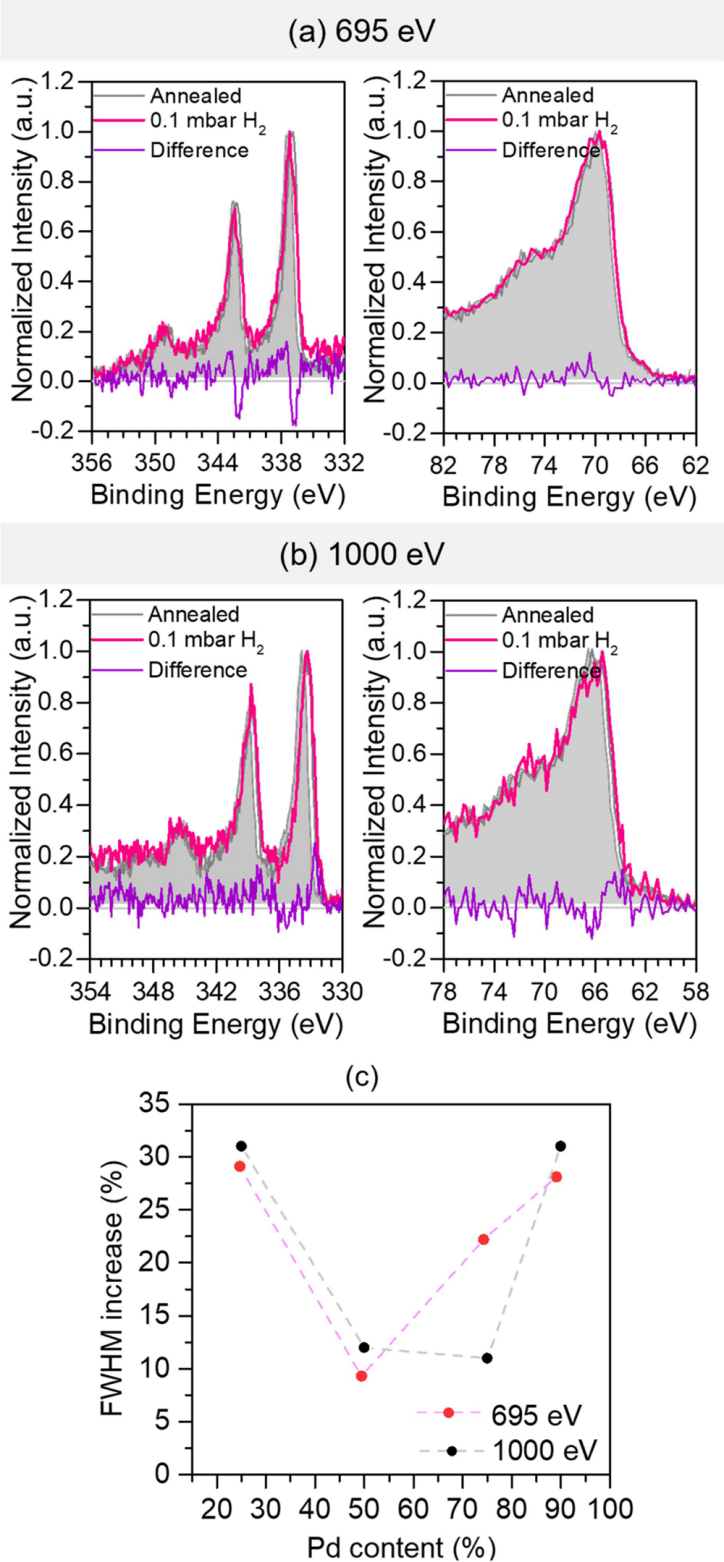
Typical AP-XPS measurements of the Pd_25_Ni_75_ nanoparticles in the Pd 3d and Ni 3p energy regions using a photon energy of (a) 695 eV and (b) 1000 eV. The gray and pink lines represent the measurement after annealing under UHV and during 0.1 mbar H_2_ exposure for 2 h at RT, respectively. The difference between the spectrum during the H_2_ exposure process and the spectrum measured after annealing is presented below each spectrum in purple. (c) Relative increase in the fwhm of the peak related to Pd(0) when comparing the Pd 3d spectra after annealing and during H_2_ exposure as a function of Pd content in the nanoparticles.

To observe the initial stages of the hydrogen storage process starting at the surface of the nanoparticles, we performed AP-XPS measurements. The initial heat treatment at 250 °C under ultra-high vacuum (UHV) facilitates organic remnant removal and the reduction of PdO to Pd(0), as demonstrated in Figure 1d. After the annealing treatment, the samples were exposed to 0.1 mbar of H_2_ while AP-XPS measurements were performed. Figure 3a,b show typical Pd 3d and Ni 3p AP-XPS measurements for the Pd_25_Ni_75_ nanoparticles using incident beam photon energies of 695 and 1000 eV, respectively. Different photon energies allow probing different depths in the nanoparticles since the inelastic mean free path (λ) of photoelectrons coming from the Pd 3d electronic region is around 13 Å (1000 eV) or 8 Å (695 eV). The spectra for all other bimetallic samples are shown in Figures S11 and S12. We carried out the measurements at RT, before and during exposure to 0.1 mbar of H_2_. The peak at around 336 eV (Figure 3a) is assigned to the Pd(0) component.^16^ For Pd_90_Ni_10_ nanoparticles, we observe that the nanoparticles are not fully reduced after thermal treatment since they show a shoulder at higher binding energies (around 338 eV), which is associated with PdO (see Figure S11a).^12^ This shoulder disappears when the sample is exposed to H_2_, thus showing the full reduction of PdO to Pd(0). The small binding energy shifts in Pd 3d and Ni 3p regions are discussed in more detail in Note S1. It is important to note that the absolute binding energy values and shifts for suspended nanoparticles are not always indicative of a chemical change. The electrostatic alignment between the particle and substrate can result in apparent shifts,^17^ as it is believed to be the case here as well. The presence of a doublet at around 349 eV is also observed, which is attributed to the Ca 2p core level that is obtained from the synthesis procedure. The main component at the Ni 3p AP-XPS region (Figure 3a) at around 70 eV comes from NiO.^18^ In this energy region, the peak at around 65 eV is associated with the Na 2s electronic region, which, similarly to Ca, is a contaminant coming from the synthesis and transfer of nanoparticles. However, no contamination was observed by EDS in these samples (see Figure S13), showing that this contamination may be small amounts on the surface of the nanoparticles.

The introduction of a H_2_ atmosphere induces an increase in the fwhm value of the Pd(0) component. The widening starts to occur at H_2_ pressures as low as 1 × 10^–7^ mbar but stabilizes at 0.1 mbar, as shown in Figure S14. Figure 3c displays the relative increase in the fwhm value of the Pd(0) component during H_2_ exposure in comparison to the value before the insertion of H_2_ atmosphere obtained from the fitting procedure performed, as shown in Figure S15 (see Table S4). All of the samples present a clear increase in the fwhm value in this period for both photon energies used. This is indicative of the presence of a second Pd component related to Pd–H bonding. However, due to the binding energy proximity of Pd(0) and Pd–H components, which is around 0.2 eV,^19^ it is not possible to distinguish the two components in the AP-XPS measurements. In almost all of the samples, this increase in the fwhm value is more significant for the measurements performed using an incident beam of 1000 eV than 695 eV. The more significant increase in the fwhm value for the higher excitation energy of 1000 eV indicates the presence of more Pd–H bonds in the subsurface layers. In general, the higher the amount of Pd in the composition, the higher the relative increase in the fwhm value. However, the Pd_25_Ni_75_ sample also presents a high relative increase due to the relatively low amount of Pd atoms that can readily form Pd–H bonds.

On the other hand, the Ni 3p AP-XPS region typically shows no changes in intensity or shape due to H_2_ exposure, thus indicating the preferential bonding of hydrogen with Pd atoms, in agreement with in situ XAS results. However, for the highest Ni amount sample (Figure 3a,b), a small increase in the fwhm of the Ni 3p region is also observed. This may indicate that hydrogen starts to be coadsorbed on Ni atoms in close proximity of Pd, which readily forms Pd–H bonds. However, this is hard to detect through the in situ XAS measurements presented because they are bulk-sensitive.

To confirm that the changes observed by in situ XAS and AP-XPS measurements are indeed due to the hydrogen storage capacity of these samples, we performed hydrogen storage measurements. For these measurements, 1 wt % of Pd_*x*_Ni_100–*x*_ nanoparticles were supported over activated carbon. The measurements were performed after exposing the samples to 1 atm H_2_ atmosphere at RT for 6 h. Figure S16 shows the hydrogen uptake as a function of the Pd concentration in the sample, and Table S5 shows the gravimetric and volumetric capacity for each sample. It is observed that the hydrogen uptake follows the same trend as the increase in the FHWM value of the Pd peak obtained from AP-XPS measurements and the increase in the Pd–Pd distance obtained from in situ XAS measurements. Thus, the changes observed in AP-XPS and in situ XAS measurements can be attributed to the hydrogen storage effect. Furthermore, the gravimetric capacity value is reproduced at least after 1 cycle of hydrogen release and hydrogen exposure again.

Since hydrogen storage occurs predominantly on the Pd atoms, it is essential to evaluate how the Pd/Ni ratio at the surface changes during the hydrogen storage process. Thus, the Pd atomic fraction at the sample surface was calculated by normalizing the Pd 4p and Ni 3p areas by their respective photoionization cross sections.^20^ The inelastic mean free path of these core–electrons was around 15 Å (1000 eV) and 11 Å (695 eV). Figure S3 shows that in all cases, Pd content at the surface is significantly smaller than that obtained by EDS. As explained above, it indicates the presence of a core–shell-like structure with a Ni-rich shell and a Pd-rich core region, even during the H_2_ exposure. Also, there is a slight increase in the Pd content at the surface region during hydrogen exposure, showing a change in the atomic arrangement of the nanoparticles.

**Figure 4:**
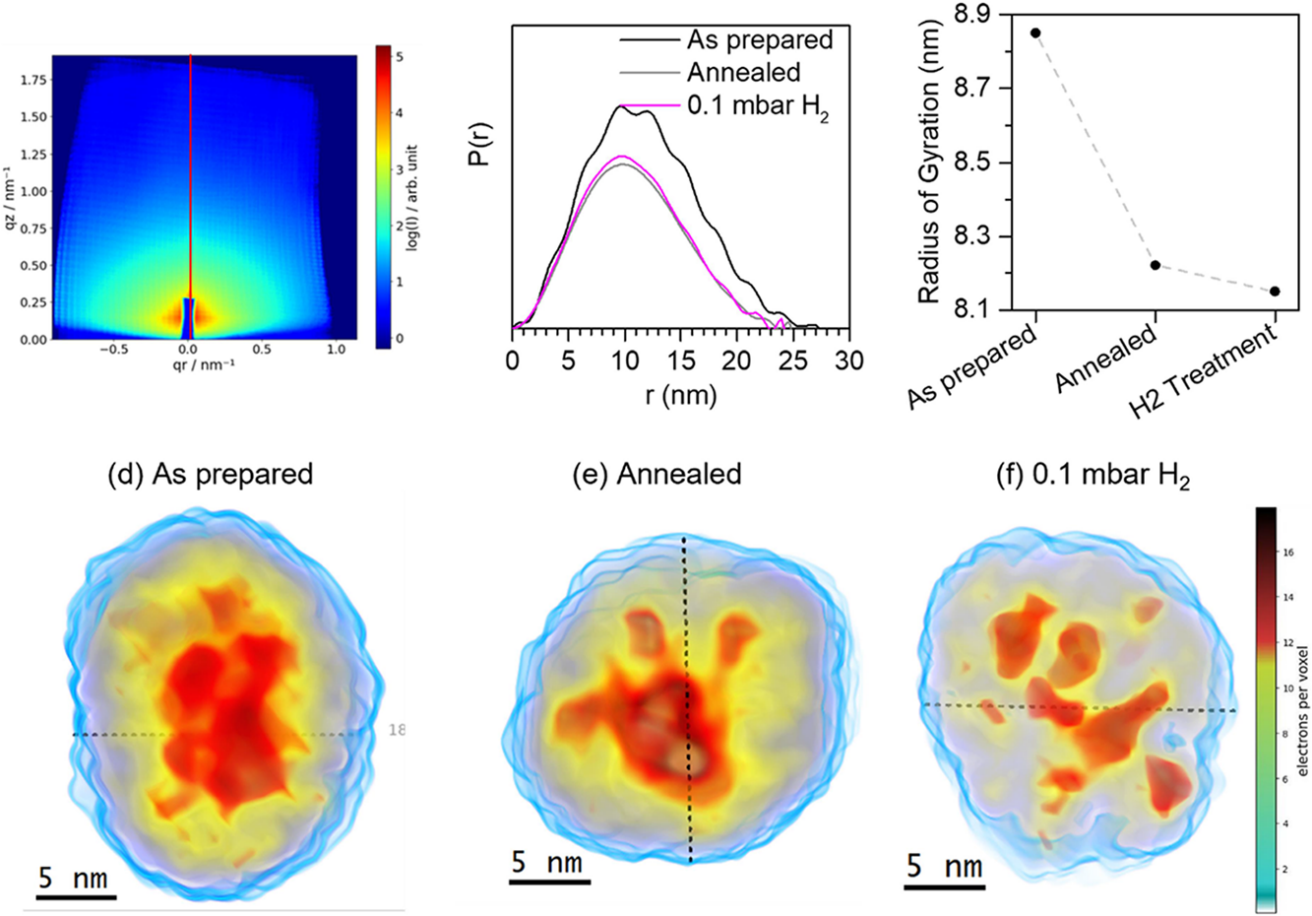
(a) Typical AP-GIXS measurement of the as-prepared Pd_25_Ni_75_ nanoparticles. The red straight line represents the position of the cut used in the analysis. (b) Pair distance distribution function (PDDF) obtained from the inverse Fourier transform of the cut presented in (A) for the Pd_25_Ni_75_ as prepared, annealed, and under a 0.1 mbar H_2_ atmosphere, presented in black, gray, and magenta, respectively. (c) Radius of gyration as a function of the treatment applied. On the bottom, reconstruction of the electronic density inside the Pd_25_Ni_75_ nanoparticles for the (d) as prepared, (e) annealed, and (f) under 0.1 mbar H_2_ atmosphere. In red is presented the region with higher electronic density, yellow is the region with medium electronic density, and blue is the region with lower electronic density.

We performed AP-GIXS measurements to gain a further understanding of the atomic rearrangement taking place in the nanoparticles during the treatment. Figure 4a presents a typical AP-GIXS measurement for the as-prepared Pd_25_Ni_75_ sample. To minimize the influence of particle clustering and island formation, we integrated over the in-plane scattering vector *q_xy_* in the region indicated by the red outline in Figure 4a to obtain the intensity as a function of the out-of-plane scattering vector *I*(*q_z_*) (Figure S20).^21^Figure 4b presents the pair distance distribution function (PDDF) obtained from the inverse Fourier transform of the line-cut. A decrease in the maximum *R*-value, which defines the maximum distance between two scattering atoms in a single nanoparticle, is observed after the annealing process. Similarly, there is a clear decrease in the Radius of Gyration (Figure 4c), which is a shape-independent indicator of particle size,^22,23^ with the treatment employed, mainly after heating to 250 °C. These results indicate the contraction of the Pd_25_Ni_75_ nanoparticles after the annealing process. This contraction can be attributed to the reduction of PdO to Pd(0).

Figure 4d–f presents the electron density reconstruction of the PDDF of the Pd_25_Ni_75_ nanoparticles under the different treatments applied (see Note S18).^24^ Pd(0) has the highest electron density of all compounds in the nanoparticles, while NiO has the lowest electron density value (Table S6). Figure 4d shows that the as-prepared Pd_25_Ni_75_ nanoparticles have an ellipsoid shape of ∼30 nm length and ∼20 nm width, which is in good agreement with the obtained AFM images of these particles (see Figure S17) and conducted GIXS simulations (see Figure S18). The particles have high electron density in the core region, while a lower density is observed in the shell region, indicating the presence of a Pd-rich core and Ni-rich shell. The size and shape of the as-prepared nanoparticles are confirmed by HAADF-STEM (Figure 1a). Figure 4e presents the PDDF reconstruction of Pd_25_Ni_75_ nanoparticles after annealing in UHV. A shape change from an ellipsoid-like to a spherical-like particle is clearly observed. Again, a higher electron density is present in the core region of these nanoparticles as compared to the shell. However, this high-density core is more diffuse in comparison to the as-prepared case. Lastly, the Pd_25_Ni_75_ nanoparticles during exposure to 0.1 mbar of H_2_ retain their spherical shape (Figure 4f). Surprisingly, the inner structure of the nanoparticle undergoes a transformation, in which segmentation of high-electron density pockets inside the nanoparticle and their diffusion toward the surface is observed during hydrogen exposition.

**Figure 5:**
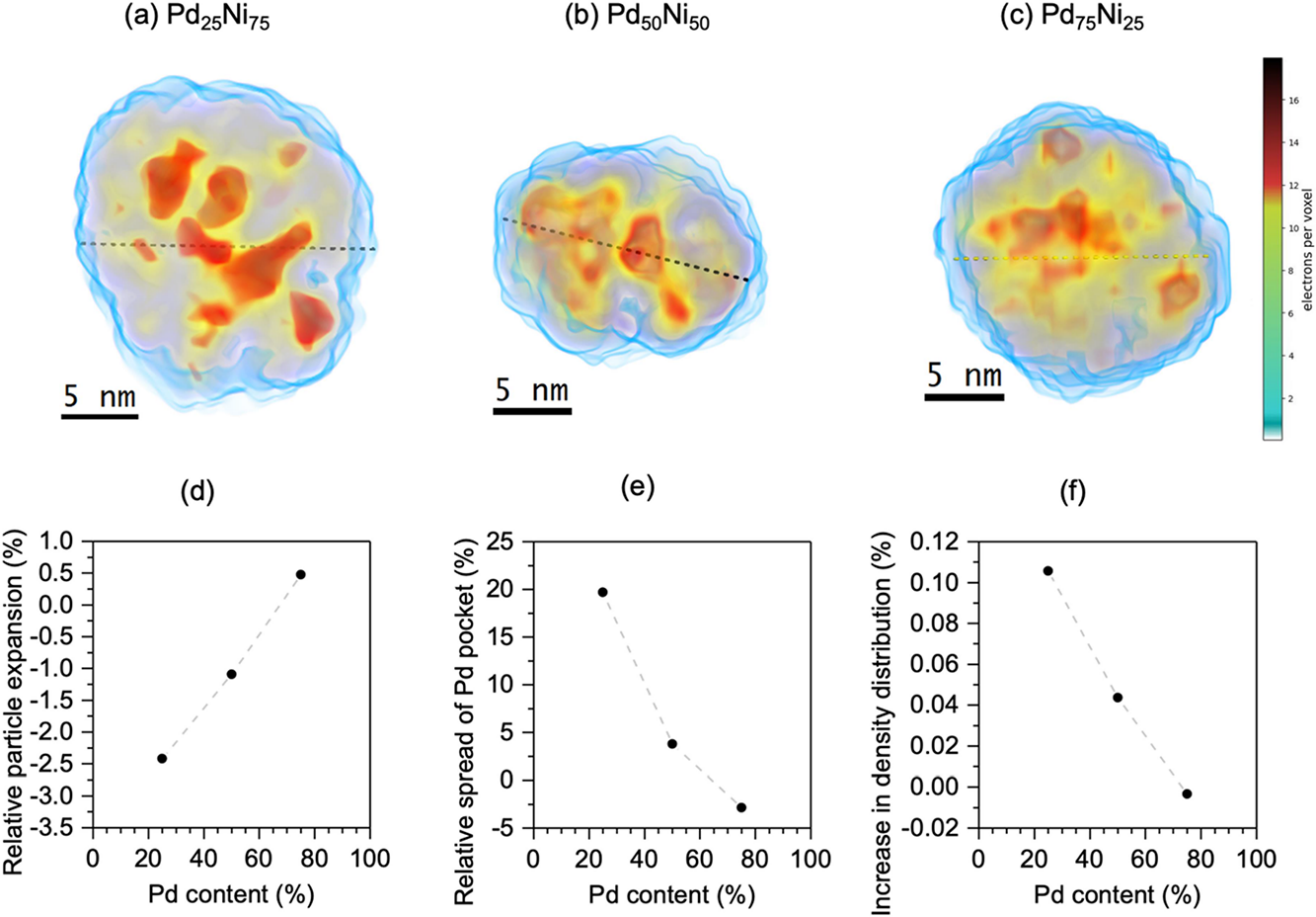
Reconstruction of the electronic density under 0.1 mbar H_2_ atmosphere for (a) Pd_25_Ni_75_, (b) Pd_50_Ni_50_, and (c) Pd_75_Ni_25_. In red is presented the region with higher electronic density, yellow, medium electronic density, and blue, lower electronic density. (d) Relative increase in particle mean diameter, calculated from the smallest particle enclosing sphere to account for the changes in shape and surface roughness during H_2_ treatment. (e) Relative expansion of the fractal enclosing sphere used to define the outer boundaries of the high density Pd core before and during H_2_ treatment in percent. (f) Relative increase in the fwhm value of the curve relative to the electronic density distribution inside the nanoparticles.

The same analysis procedure was applied to the other Pd_*x*_Ni_100–*x*_ compositions, as shown in Figure 5a–c for 0.1 mbar of H_2_ exposure. The same qualitative behavior is observed for all three stoichiometries (see Figure S19 for the annealed sample), i.e., the core region with a higher electron density in comparison to the shell region and the formation of fragmented high electronic density pockets. It explains the small intensity at FT for the Pd K edge and the same intensity for the Ni K edge in comparison to Pd(0) and NiO standards (Figure 2).

Figure 5d shows the relative expansion of the nanoparticles during the exposure to 0.1 mbar of H_2_ in comparison to before H_2_ introduction as a function of Pd content. A contraction of the Pd_25_Ni_75_ and Pd_50_Ni_50_ nanoparticles is observed, while the Pd_75_Ni_25_ nanoparticles undergo an expansion during H_2_ exposure. The expansion is related to the increase in the Pd lattice size when hydrogen is adsorbed in the interstitial site, as determined by in situ XAS measurements. However, due to the low amount of Pd and the complicated rearrangement of the Pd pockets inside the nanoparticles, Pd_25_Ni_75_ and Pd_50_Ni_50_ nanoparticles exhibit a size contraction.

Moreover, Figure 5e shows the relative expansion of the sphere enclosing all of the high-electron density pockets as a function of the Pd content in the sample. Such analysis utilizing an equivalent sphere is typically used for fractal nanoparticle systems to compare different shape configurations.^25^ For lower Pd concentrations, a pocket enclosure expansion of 20% is observed. This occurs by the dissolution of high electron density regions inside the nanoparticles with a lower Pd content. A contraction occurs for nanoparticles containing a higher Pd concentration. Figure 5f shows the increase in the fwhm of the electronic density distribution curve across the particle, as shown in Figure S20.

**Figure 6:**
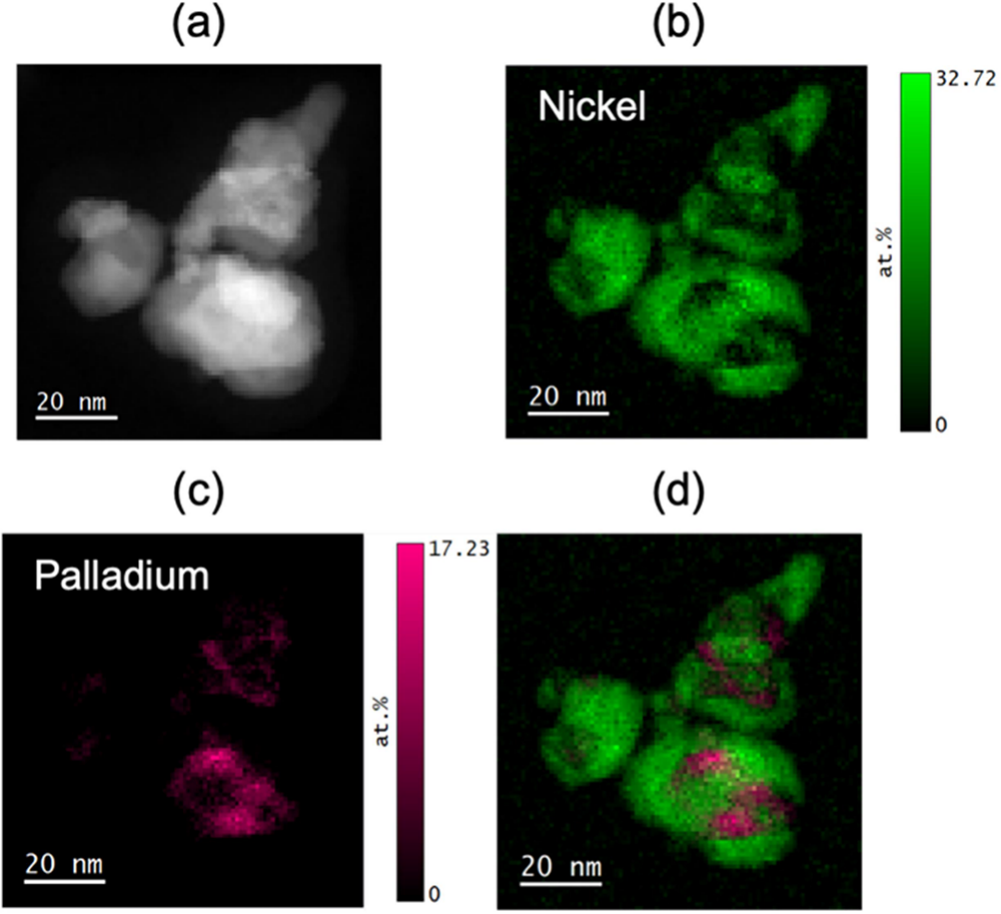
(a) STEM-HAADF image of a typical as prepared Pd25Ni75 nanoparticle from which (b) the Ni compositional map was obtained by integration of the L edge and (c) the Pd compositional map was obtained by integration of the M edge. (d) A composite map overlapping the Ni (green) and Pd (magenta) maps. The color bars in these images show the atomic percentage which is calculated based on all the available elements in the spectral window from 230 to 1419.3 eV, which includes both C K-edge (∼284 eV) and O K-edge (∼532 eV).

To validate the conclusions obtained from the AP-GIXS analysis, STEM-EELS measurements were performed. Figure 6 shows a typical STEM-HAADF image of the as-prepared Pd_25_Ni_75_ nanoparticles with around ∼20 nm diameter and its Pd and Ni compositional maps (see Figure S21 for a typical EELS spectrum). The Ni composition maps (Figure 6b) clearly show pockets of Ni-deficient regions in the nanoparticles. These deficient regions are also observed in the O composition map obtained by integrating the O K edge due to the atomic density difference of PdO and NiO (shown in Figure S22). The Pd compositional map (Figure 6c) shows the presence of Pd-rich clusters of around 3–10 nm in size located in the Ni deficient regions. A composite map formed by overlaying the Ni (green) and Pd (magenta) compositional maps is shown in Figure 6d. It is evident that multiple pockets of Pd-rich clusters are embedded in the NiO matrix of a single nanoparticle, as validated with several other nanoparticles (Figure S23).

All of the as-prepared Pd_*x*_Ni_100–*x*_ nanoparticles exhibit an internal structure with a Pd-rich core and Ni-rich shell, as indicated by the compositional difference between bulk-sensitive EDS and surface-sensitive XPS analysis. This phase separation was confirmed by AP-GIXS (Figure 4d) and EELS measurements (Figure 6). In addition, it was observed that the as-prepared samples present mainly NiO and PdO compounds. For the metallic Pd–Ni system, the opposite is theoretically expected, i.e., a Pd-rich shell and a Ni-rich core^26^ but the Ni-rich surface comes due to the stability of NiO. Upon annealing under UHV or exposure to H_2_ at RT, Pd in these nanoparticles undergoes reduction from PdO to Pd(0). On the other hand, from the AP-XPS and in situ EXAFS measurements (Figures 2 and 3) it is evident that Ni remains as NiO, which agrees with previous reports.^27^ In addition, during reduction under a H_2_ atmosphere at RT, we observed that the sample with a higher amount of Ni presented a slower reduction than the sample with a higher amount of Pd. This can be related to the existing core–shell-like structure. The Ni-richer samples present a thicker (and consequently more impermeable) shell, acting as a diffusion barrier for the H_2_ molecules. It makes the reduction kinetics of PdO slower due to the slower diffusion of the H_2_ molecules through the Ni shell, which is a necessary step to reach the Pd-rich core. The opposite is observed during the reduction process under UHV. In this case, since the thermally induced reduction process does not depend on the diffusion of H_2_ molecules through the NiO layer, the Ni-rich sample reduces at a lower temperature than the Pd-rich sample.

During exposure to a H_2_ atmosphere, the EXAFS measurements showed an increase in the Pd–Pd interatomic distance (Figure 2c), while no difference is observed in the Ni K-edge. From the d-band theory,^28^ it is known that the strain influences the hydrogen storage properties. Lattice expansion upshifts the d-band center, leading to stronger adsorbate binding. The increase in the Pd–Pd interatomic distance may also be related to the storage of hydrogen in the interstitial sites around Pd atoms, thus increasing the lattice size, as observed in the literature.^29,30^ Similarly, we observed an increase in the fwhm of the Pd(0) (Figure 3c) peak at Pd 3d AP-XPS measurements under a H_2_ atmosphere. This increase in the fwhm is also attributed to the storage of H atoms on Pd, since, as explained before, it indicates the presence of a second Pd component (Pd–H). In both cases, no change is observed in the Ni K-edge or Ni 3p peak spectra. Thus, it shows that the hydrogen atoms get exclusively adsorbed on the Pd atoms. In addition, it is observed that the highest increase in the R-value and fwhm increase the highest Pd content on the studied stoichiometries. Thus, it is expected that the Pd_90_Ni_10_ sample would have the highest hydrogen storage capacity, which was confirmed by hydrogen storage measurements. Intuitively, although Pd_75_Ni_25_ and Pd_25_Ni_75_ present a similar increase in the R value and fwhm, the former is expected to exhibit a higher hydrogen storage capacity due to the higher Pd content. However, we observed a similar gravimetric capacity for the two samples. This may be related to a higher propensity for phase separation of the Pd as observed by AP-GIXS measurements. Therefore, both the Pd–Pd distance measurements (Figure 2c), relative fwhm increase of the Pd(0) peak at Pd 3d spectra (Figure 3c), and gravimetric capacity show a relative minimum at the Pd_50_Ni_50_ composition.

Moreover, it was previously observed that 1 wt % of Pd nanoparticles supported on activated carbon present a storage capacity of around 0.7 wt %.^31^ In the current study, we show that the addition of only 25% Pd gives the same hydrogen storage capacity. The hydrogen storage capacity of the activated carbon alone was also measured, and it is around 0.08 wt %. Thus, the bimetallic nanoparticles (with the exception of Pd_50_Ni_50_) present an increase in the gravimetric capacity by more than a factor of 8. On the other hand, Pd_50_Ni_50_ nanoparticles show a reduced hydrogen storage of 0.4 wt %, which is in line with previous findings.^11^ Since Pd is a much more expensive resource compared to Ni, it is important to highlight the economic importance of these results. Adding a small amount of Pd (∼25%) is enough to have a hydrogen uptake value similar to the monometallic Pd nanoparticles. It indicates that such high Ni-content nanoparticles can provide an economically viable solution for hydrogen storage.

The phase segregation observed by AP-GIXS (Figure 4) is induced by the storage of hydrogen mainly onto the Pd atoms. The interchange of atoms in a core–shell structure under a reducing or oxidizing atmosphere has been widely observed for many different bimetallic systems at elevated temperatures.^32^ However, the interchange of atoms from the Pd core into the nanoparticle shell at RT, as observed here, causes the core to split into Pd pockets inside a Ni shell. In addition, the formation of well-defined high-density structures is observed. To the best of our knowledge, this is the first time the segregation of the core of the nanoparticle into small pockets has been observed in situ induced by exposure to hydrogen at room temperature.

In the samples with a higher Pd content, the Pd is more dispersed inside the nanoparticles, as observed in the AP-GIXS measurements (Figure S17). This might be due to either the lack of NiO in the nanoparticles to form a proper core–shell structure or the formation of a solid solution. Theoretical ternary Ni–Pd–O convex hull drawn using the Materials Project (Figure S22) does show Pd_50_Ni_50_ (∼43 meV) close to the stable convex hull.^33,34^ Previous work has shown that such structures might get stabilized at higher temperatures.^35,36^ This stabilization is due to entropic effects which depend critically on how the target alloy composition of interest responds to the temperature effects in contrast to all relevant competing phases.^37,38^ However, to the best of our knowledge, experimental Ni–Pd–O phase diagrams have not been reported to date.

The effect of the phase separation can be visualized by the reconstructed electronic density obtained from the AP-GIXS measurements (Figures 5a and S17). If the Pd_50_Ni_50_ composition is stabilized and forms a solid solution, the Pd_75_Ni_25_ composition would phase separate into pure Pd-rich and Pd_50_Ni_50_ regions, thereby explaining the presence of very high Pd-rich regions. However, for both Pd_50_Ni_50_ and Pd_25_Ni_75_ samples, no Pd-rich regions are observed. This is most likely because these compositions are either stable (the case of Pd_50_Ni_50_) or the phase separate in the stable Pd_50_Ni_50_ and Ni-rich compositions (the case of Pd_25_Ni_75_).

From the AP-GIXS measurements, it is evident that during the hydrogen storage process, considerable rearrangement of Pd atoms is observed. In general, upon H_2_ storage, the increase in particle size is in proportion to the Pd content of the nanoparticle. However, the relative Pd pocket expansion and relative distribution compared to the annealed nanoparticles decrease with the increase in the Pd content under the H_2_ atmosphere. This is most likely due to the lack of a well-delineated Pd core in high Pd-containing samples. However, in all cases the hydrogen storage leads to a rearrangement of Pd, forming Pd-rich pockets.

Previously, evidence of the atomic rearrangement during the hydrogen storage process was also observed in a Pd-rich core and Pt-rich shell nanoparticles. Tayal et al.^39^ concluded that the storage of hydrogen in this sample transformed the core–shell structure into a mixed Pd–Pt alloy. This process increased the Pd–Pt interfacial area, where the hydrogen was adsorbed, and then increased the hydrogen storage capacity of this sample. Similarly, in this work, the sample with the smallest amount of Pd presents many small Pd pockets that are very well delimited and segregated from the NiO phase. This process increases the interfacial area between the Pd and NiO phases where the hydrogen can be stored.^7,39,40^

In fact, a deeper look at the Ni K edge XANES spectra (Figure S23) shows changes in the edge position. In all samples, the introduction of H_2_ induces a shift to smaller binding energies, as observed elsewhere.^39^ It indicates that NiO is not just a bystander during the hydrogen storage process but the hydrogen is adsorbed at the interface between the NiO shell and Pd pockets. It compensates for the small amount of Pd in these nanoparticles, leading to strong changes in the parameters related to hydrogen storage from AP-XPS measurements. On the other hand, in Pd-rich nanoparticles, aside from starting to form Pd pockets, this structure is not clearly defined, presenting a diffused transition between Pd pockets and NiO shell. These results indicate that the hydrogen storage process occurs efficiently in these Pd pockets, where a plum pudding-like system is formed. It also reveals the underlying mechanisms, which can be used to reduce the use of noble metals while retaining the H_2_ storage capacity in bimetallic nanoparticles. This could also be a possible explanation for the similar gravimetric capacity observed for the Pd_25_Ni_75_ and Pd_75_Ni_25_ samples. Considering this, the main mechanism of hydrogen storage in the Pd–Ni nanoparticles consists of the hydrogen absorption at the interface of Pd pockets and NiO medium.

## Conclusions

In this work, we explored the behavior of Pd_*x*_Ni_100–*x*_ core–shell nanoparticles during hydrogen storage, revealing intriguing transformations in both their surface and internal structure. By employing advanced in situ X-ray techniques, we gained a comprehensive understanding of how these nanoparticles change morphologically and chemically at the atomic level. While hydrogen primarily interacts with Pd, NiO plays a crucial role by acting as a buffer that allows Pd to form internal pockets. As hydrogen is adsorbed, these pockets fragment, significantly increasing their surface area available for hydrogen storage, even if the overall particle size decreases. Notably, samples with lower Pd content exhibit hydrogen uptake similar to pure Pd, showcasing that small addition of Pd (∼25%) to the NiO system presents great potential to reduce costs in hydrogen storage technologies while maintaining or increasing the performance. Our insights also suggest that future designs of bimetallic nanoparticles should focus on enhancing the formation of hydrogen-active nanopockets of material, paving the way for more efficient and cost-effective hydrogen storage solutions. A deeper understanding of hydrogen storage mechanisms, as presented here, will drive the development of sustainable energy technologies necessary for the transformation of the energy industry.

## Methods and Experimental Section

### Synthesis

We synthesized Pd_*x*_Ni_100-x_ nanoparticles with different compositions, namely Pd_90_Ni_10_, Pd_75_Ni_25_, Pd_50_Ni_50_, and Pd_25_Ni_75_. The total mass of nickel chloride hexahydrate plus palladium acetate was kept constant at 92.9 mg in all cases, and its individual values were chosen to achieve the desired Pd:Ni atomic ratios. Initially, nickel chloride hexahydrate (NiCl_2_·6H_2_O) and glucose (0.039g) were totally dissolved in Milli-Q water (7.2 mL), while palladium acetate (Pd(OCOCH_3_)_2_) was dissolved in monoolein (1-Oleoyl-rac-glycerol technical grade, ∼40% purity) (20 g). This monoolein phase was kept in an ultrasonic bath for 5 min while it was constantly stirred with a spatula. The aqueous phase was added to the monoolein phase and mixed for 1 min using a spatula. This mixture rested for 10 min. After this, it was added to a water bath at 80 °C for 1 h and mixed with a spatula every 5 min. After the bath, the mixture rested for 2 h, and then they were calcined under air at 500 °C for 4 h, forming a fine dark powder containing the nanostructures.

### Transmission Electron Microscopy (TEM)

We performed TEM measurements at CM-UFMG using a Tecnai G2 Spirit Biotwin microscope operated at 120 kV. For the measurements, the samples’ powder was dispersed in Milli-Q water and left in the ultrasound bath for 15–20 min. Then, three drops of this solution were added to a carbon-coated Cu grid. The size distribution histograms of the Pd_*x*_Ni_100–*x*_ nanoparticles were obtained by using ImageJ (Version 6.0) software.^200^ The diameter of each nanoparticle was calculated from the total area of its projection on the TEM screen, considering spherical nanoparticles.

### Electron Energy Loss Spectroscopy (EELS)

We performed EELS measurements using a TEAM 1 microscope (double aberration-corrected Thermo Fisher Scientific Titan 80-300) at the National Center for Electron Microscopy (NCEM). The EELS data set was collected at 300 kV using a Gatan Continuum spectrometer, a convergence angle of approximately 30 mrad, and a collection angle of 110 mrad. The width of the zero-loss peak was measured to be 1.2 eV, and a 0.35 eV/channel dispersion was used to collect the spectra. The composition map was obtained by integrating the Ni L edge (*L*_2_ ∼ 872 eV and *L*_3_ ∼ 855 eV), Pd M edge (*M*_4_ ∼ 340 eV and *M*_5_ ∼ 335 eV), and O K edge at 532 eV.

### X-ray Diffraction (XRD)

We performed the XRD measurements in a Rigaku Ultima IV diffractometer at CNANO-UFRGS using Cu Kα (1.5406 Å) radiation with a graphite monochromator working at 40 kV and 17 mA. Pd_*x*_Ni_100-x_ samples were fixed in a glass blade for the XRD measurements. The data were collected for a 2θ range between 20° and 100°, using a scanning step of 0.05°. Pattern indexing was performed using reference standards obtained from the ICSD database.^41^ The Rietveld refinement was performed on FullProof^42^ software (version July 2017). The Instrumental Resolution Function (IRF) was obtained from a SiO_2_ standard. Rietveld refinements were carried out using the pseudo-Voigt profile function of Thompson, Cox, and Hastings^43^ and a linear background. During the refinement, the Debye–Waller overall factor and the atom occupancy were fixed to the crystallographic information file (CIF) value obtained from the literature.

### In Situ X-ray Absorption Spectroscopy (XAS)

We performed in situ XAS measurements at the NOTOS beamline from ALBA Synchrotron Light Source in transmission mode at the Pd K edge (24350 eV) and Ni K edge (8333 eV). For the measurements, the nanoparticle powder was supported over activated carbon (Activated Charcoal Norit) with 7 wt % using an ultrasound bath for 20 min. For the Pd K edge and Ni K edge measurements, 13 mm pellets were produced with 75 mg of Pd_*x*_Ni_100-x_/C and 25 mg of BN. For the measurements, the samples were initially exposed to 30 mL/min N_2_ and heated to 150 °C where they remained for 15 min. Then, the nanoparticles were cooled to room temperature (RT) under 30 mL/min He. At RT, the samples were exposed to 30 mL/min of 4% H_2_ + 96% He and remained in this condition until its complete stabilization, i.e., until no more changes in the X-ray absorption near edge structure (XANES) spectra were observed. The XAS measurements were performed during the full treatment employed. The XANES and EXAFS measurements for the Ni K edge were performed by integrating 0.24 s/point and with a 0.93 and 1 eV energy step, respectively, while for the Pd K edge, the integration time was 0.2 s/point and the energy step used was 0.5 and 1 eV, respectively. The Extended X-ray Absorption Fine Structure (EXAFS) spectra were analyzed in accordance with the standard procedure of data reduction^44^ using IFEFFIT.^45^ The EXAFS signal χ(*k*) was extracted and then Fourier transformed using a *k*^2^-weighted χ(*k*) and a Kaiser-Bessel window with a *k*-range of 10.0 Å^–1^ for both edges. The phase shift and amplitudes were obtained with the FEFF6 code by using an fcc metallic Pd, tetragonal PdO, fcc metallic Ni, and a fcc NiO cluster with a 6 Å radius each. For the fit of the EXAFS oscillations, the amplitude reduction factor (S0_2_) was fixed at 0.85, as obtained from the fit of both the Pd(0) and Ni(0) standards. The *R*-factor obtained from the analysis was always lower than 0.01, which demonstrates the excellent agreement between the proposed model and the experimental result.

### Ambient Pressure X-ray Photoelectron Spectroscopy (AP-XPS) and Ambient Pressure Grazing Incidence X-ray Scattering (AP-GIXS)

We performed AP-XPS and AP-GIXS measurements at the APPEXS endstation at the 11.0.2 beamline of ALS Synchrotron.^46^ For the measurements, the nanoparticle powder was dispersed in ethanol and added to an ultrasonic bath for 10 min. After that, a silicon wafer was covered three times with the solution containing the nanoparticles, thus forming a thin layer. After that, the samples were inserted into an ambient pressure reaction chamber, where they were heated to 250 °C under vacuum to clean the sample’s surface and to reduce from PdO to Pd(0). Then, the samples were cooled to room temperature, and once at this temperature, they were exposed to 0.1 mbar H_2_. AP-GIXS measurements were performed for the samples as prepared, after annealing, and during hydrogen exposure. AP-XPS measurements were performed before and after each AP-GIXS measurement. For the AP-XPS measurements, a SPECS PHOIBOS 150 NAP analyzer was used, with a photon beam energy of 1000 and 695 eV. Energy regions collected were survey, Pd 3d, Ni 3p, Pd 4p, Si 2p, C 1s, and O 1s. A step of 1 and 0.1 eV, dwell time of 0.2 s, and pass energy of 10 eV were used for the survey and high-resolution spectra, respectively.

KOLXPD software (version 1.8.0) was used for the XPS analysis. For the fitting of the high-resolution spectra, a Shirley-type background was used. The fitting procedure, shown in Figure S15, was performed by fixing the distance between the Pd(0) and PdO and Pd(0) satellites at 1.3^47^ and 6.7 eV,^48^ respectively. An asymmetric Doniach Sunjic peak was used for the fit of the Pd(0) component, and Voigt peaks were used for all of the other chemical components. The Gaussian width of the peaks was fixed at the same value for all of the peaks. The Lorentzian width was fixed for the same component in all of the measured conditions, except for the Pd(0), where asymmetry was fixed.

For the AP-GIXS measurements, a beam energy of 1240 eV, corresponding to a wavelength of 1 nm, was used. The total collection solid angle was ±12° in-plane and 24° out of plane, which translates to *q*_max_ ∼ 0.9 and 1.8 nm^–1^, respectively. Andor iKon-L CCD mounted on the biaxial quasi-spherical manipulator was used to collect scattered photons. To minimize the influence of island formation and particle clustering, GIXS analysis was performed on an out-of-plane line-cut extracted at a scattering vector (*q*) of 0.0 nm^–1^.^49^ The PDDF was obtained from the inverse Fourier transform of the horizontal line cut utilizing the BioXTAS RAW software package (see Note S18 for details on GIXS line cuts and analysis).^50−52^ The electron density reconstruction was performed utilizing the BIOXTAS RAW and the integrated DENS package (details on the reconstruction are found in Note S2).^24^

We also performed AP-XPS measurements at the APXPS endstation at the 9.3.2 beamline of ALS Synchrotron. The nanoparticles were prepared in the same way as presented for the measurements at 11.0.2. The nanoparticles were inserted into an ambient pressure chamber and heated to 700 °C under a vacuum. Pd 3d and Ni 3p energy regions were measured during the heating process. After that, the nanoparticles were cooled down to room temperature where they were exposed to 1 × 10^–7^, 1 × 10^–5^, 1 × 10^–3^, 1 × 10^–1^, and 5 × 10^–1^ mbar of H_2_. At each pressure, energy regions collected were survey, Pd 3d, Ni 3p, Pd 4p, Si 2p, C 1s, and O 1s. All the measurements were performed using a 695 eV photon beam energy, a pass energy of 20 eV, a dwell time of 100 ms, and step sizes of 0.1 eV and 0.5 were used for the high-resolution and survey spectra.

### Hydrogen Storage Capacity Measurements

We determined the hydrogen storage capacity by Gas Chromatography (GC) measurements at the Physics of Nanostructures Laboratory–UFRGS using a GC Model 310 USB from SRI Instruments. The GC equipment is equipped with a Thermal Conductivity Detector (TCD) and operates with Ar as the carrier gas. A home-built reactor was used for the reactions. Initially, the Pd_*x*_Ni_100-x_ nanoparticles were supported over activated carbon (Activated Charcoal Norit) with 1 wt % through mechanical support followed by an ultrasound batch for 20 min. A sample of pure activated carbon was also measured for comparison purposes. The sample was introduced into the reactor, and it was purged with Ar before inserting a 1 atm Ar atmosphere. After this, the system was heated to 150 °C and remained at this temperature for 1 h to remove humidity and to clean the nanostructure surface. Then the system was purged with Ar and cooled to room temperature under a 1 atm Ar atmosphere. After the thermal treatment, 1 atm H_2_ was inserted in the reactor, and aliquots of 150 μL of the gas atmosphere inside the reactor were taken every 60 min to determine the H_2_ amount in the inner atmosphere of the reactor by GC measurements. The comparison with the initial amount of H_2_ enables us to determine the amount of H_2_ adsorbed by the sample. This measurement was repeated until stabilization of the H_2_ amount inside the reactor, which occurred after around 6 h. At the end, the sample was heated to 350 °C for 1 h for H_2_ release and thus it was exposed to the same conditions for a new measurement of H_2_ storage.

The gravimetric capacity was calculated using
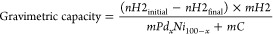
where nH2 is the number of moles of H_2_ calculated from the amount of H_2_ present in the gas of the reactor considering it as an ideal gas and m stands for the mass. The pressure and temperature inside the reactor were constantly monitored with the use of a sensor, and it remained close to 1 atm and 20 °C, but small differences observed were also considered in the determination of the gravimetric capacity.

### Scanning Electron Microscopy (SEM) and Energy Dispersive Spectroscopy (EDS)

We obtained the SEM images and EDS spectra at the Molecular Foundry-LBL using a Zeiss Gemini Ultra-55 microscope operated at 5 kV. The samples were measured before and after the AP-XPS and AP-GIXS measurements. The SEM images were obtained by detecting secondary electrons.

### Atomic Force Microscopy (AFM)

We conducted AFM measurements at the Imaging Facility of the Molecular Foundry-LBL using an Asylum Jupyter AFM and a PEAKFORCE-HIRS-F-A tip from BrukerNano. The AFM was operated in tapping mode, with a drive amplitude of 100 mV and a set point of 60 mV. The scan speed was set to 0.75 lines per second and the resolution to 256 × 256 lines × rows.

## Supporting Information Available

TEM, XPS, EXAFS, EDS, gravimetric capacity, AFM, GIXS, and EELS data, and analysis (PDF)
