# Supplementary material for: Direct Observation of Phase Change Accommodating Hydrogen Uptake in Bimetallic Nanoparticles
Source: ACS Nano. 2025 Mar 5;19(10):10312–22. doi: 10.1021/acsnano.4c18013 (PMC11924317; doi:10.1021/acsnano.4c18013)
Supplement: Supplementary file 1 — nn4c18013_si_001.pdf [file nn4c18013_si_001.pdf]

Supporting Information

Direct observation of phase change accommodating hydrogen uptake in bimetallic nanoparticles

Lívia P. Matte,[1,2] Maximilian Jaugstetter,[3] Alisson S. Thill,[1] Tara P. Mishra,[4] Carlos Escudero,[5] Giuseppina Conti,[2,6] Fernanda Poletto,[7] Slavomir Nemsak,[2,6] Fabiano Bernardi[1*]*

[1] Programa de Pós-Graduação em Física, Instituto de Física, Universidade Federal do Rio Grande do Sul (UFRGS); Porto Alegre, 91501-970, Brazil.

[2] Advanced Light Source, Lawrence Berkeley National Laboratory; Berkeley, 94720, USA.

[3] Chemical Sciences Division, Lawrence Berkeley National Laboratory; Berkeley, 94720, USA.

[4] Materials Science Division, Lawrence Berkeley National Laboratory; Berkeley, 94720, USA.

[5] ALBA Synchrotron Light Source, Cerdanyola del Vallès; Barcelona, 08290, Spain.

[6] Department of Physics and Astronomy, University of California; Davis, 95616, USA.

[7] Departamento de Química Orgânica, Instituto de Química, Universidade Federal do Rio Grande do Sul (UFRGS); Porto Alegre, 91501-970, Brazil.

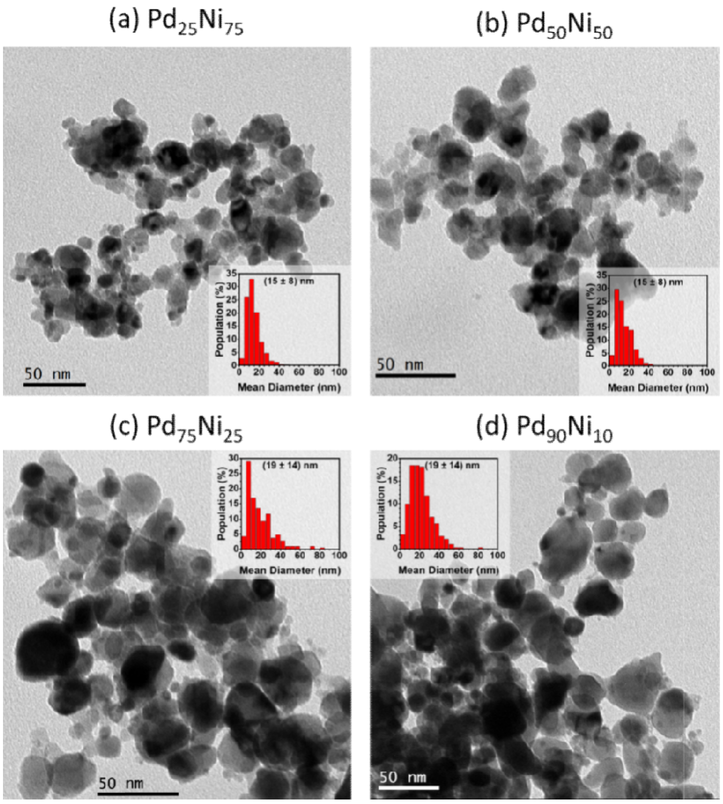

Fig. S1: Typical TEM images of the (a) $Pd_{25}Ni_{75}$, (b) $Pd_{50}Ni_{50}$, (c) $Pd_{75}Ni_{25}$, and (d) $Pd_{90}Ni_{10}$ nanoparticles. The inset shows the histogram of size distribution.

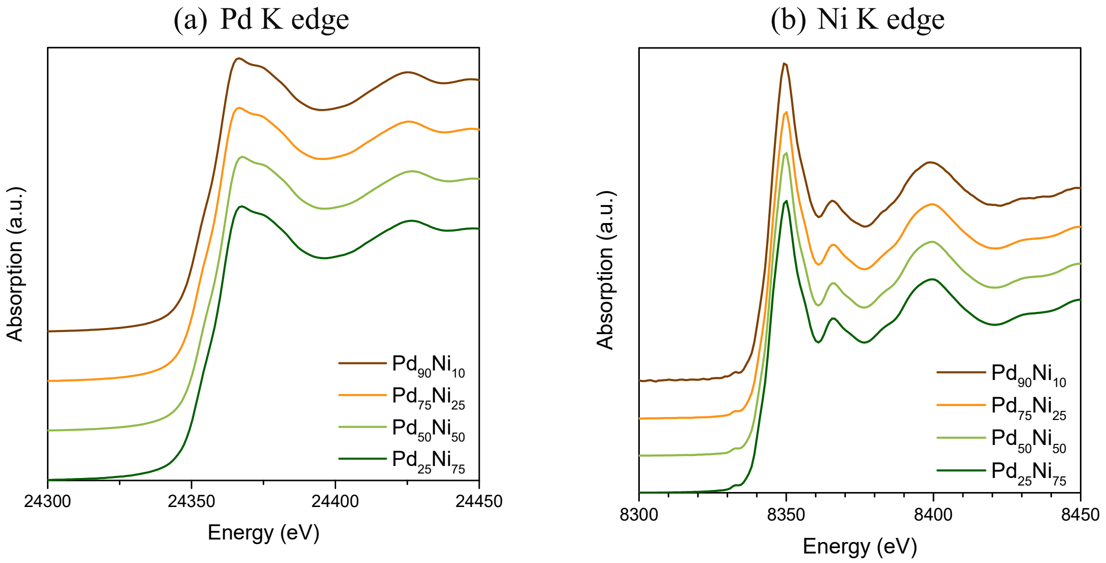

Fig. S2: XANES measurements of the as-prepared samples at the (a) Pd K edge and (b) Ni K edge.

Table S1: Comparison between the nanoparticles mean diameter obtained from the TEM imagens and the crystallite size obtained from the XRD diffractograms.

	**XRD (crystallite size)**			**TEM**
	**NiO**	**PdO**	**Pd**	**(mean diameter)**
$Pd_{25}Ni_{75}$	18 nm	19 nm		(15 ± 8) nm
$Pd_{50}Ni_{50}$	8 nm	13 nm		(15 ± 8) nm
$Pd_{75}Ni_{25}$	20 nm	26 nm	29 nm	(19 ± 14) nm
$Pd_{90}Ni_{10}$		23 nm	33 nm	(19 ± 14) nm

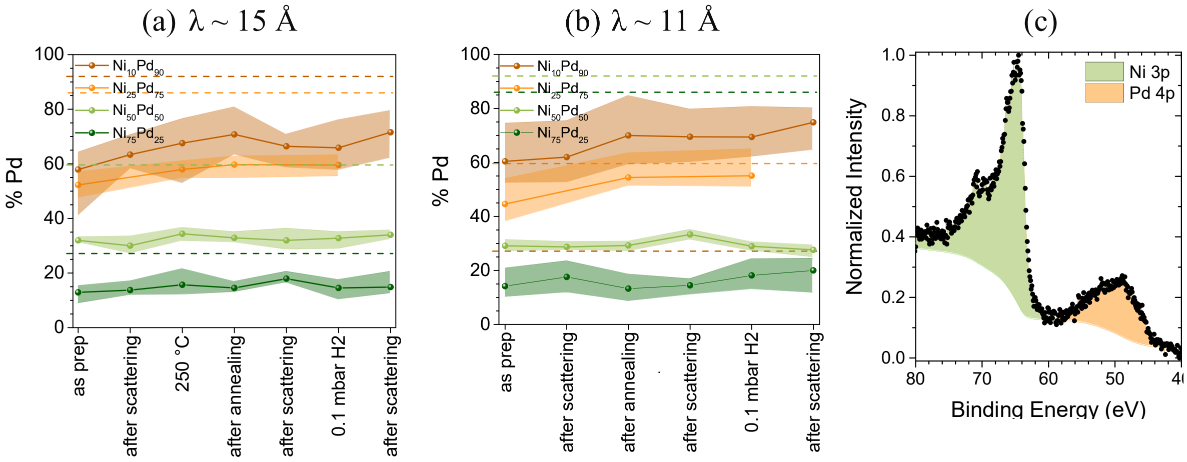

Fig. S3: Pd percentage in the surface of the nanoparticles considering two different depths probed with (a) λ ~ 15 Å (hv = 1000 eV) and (b) λ ~ 11 Å (hv = 695 eV) obtained from the shaded area presented in (c), where the orange and green areas represent the Pd 4p and Ni 3p energy regions, respectively. The main lines in Fig. (a) and (b) represent the mean value obtained after 5 analysis process, while the shaded areas include all the values obtained. The brown line represents the value obtained for the $Pd_{90}Ni_{10}$, orange represents the $Pd_{75}Ni_{25}$, light green represents the $Pd_{50}Ni_{50}$, and dark green represents the $Pd_{25}Ni_{75}$ nanoparticles. The dashed lines represent the value obtained by EDS.

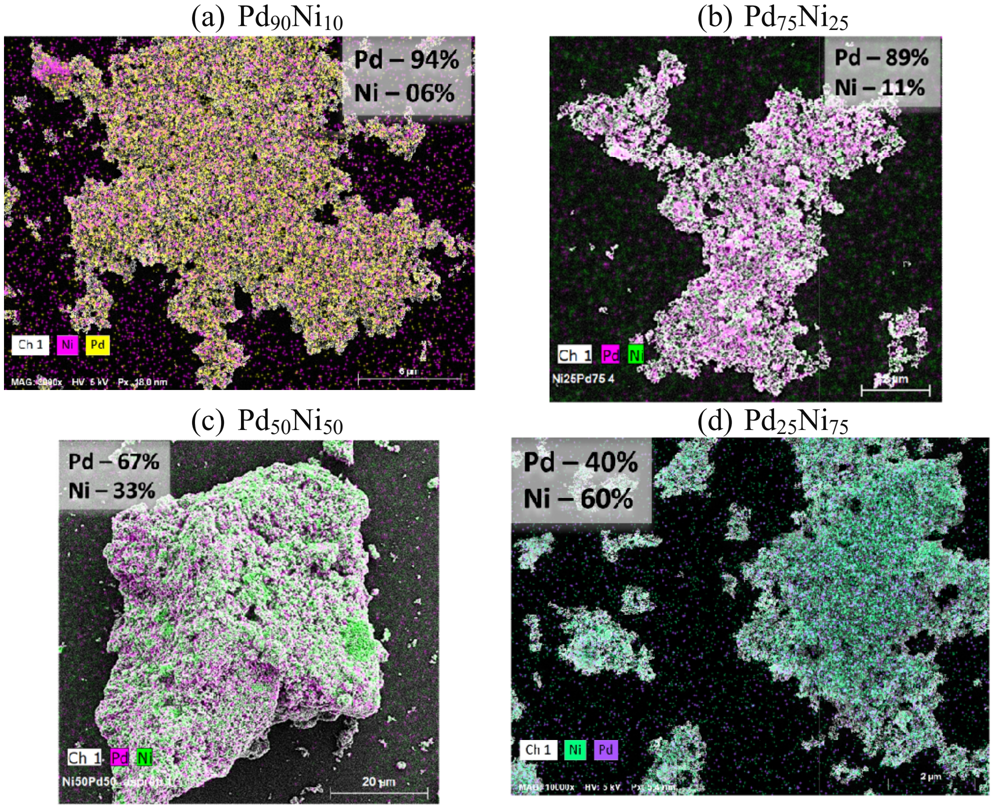

Fig. S4: EDS maps of the different Ni_xPd_{100-x} bimetallic nanoparticles studied. The Pd and Ni fraction for each sample is shown in the inset.

Note: Fig. S5 shows the time evolution of the XANES spectra measured at room temperature while the samples were exposed to 30 mL/min 4% H_2 + 96% He. Initially, all the samples present one intense peak after the absorption edge, which is characteristic of PdO XANES spectrum. During the exposure to H_2 atmosphere, most of the samples evolve and their XANES spectra present a change from one to two peaks near the absorption edge. These two peaks are characteristic of a Pd(0) XANES spectrum, then indicating the samples' reduction. However, this evolution is not observed for the sample with the highest amount of Ni ($Pd_{25}Ni_{75}$), which still exhibits only one peak after the absorption edge, indicating a high amount of PdO in this sample. In addition, the samples present different reduction times depending on the Pd fraction in the nanoparticles (the higher the Pd content the fastest the reduction).

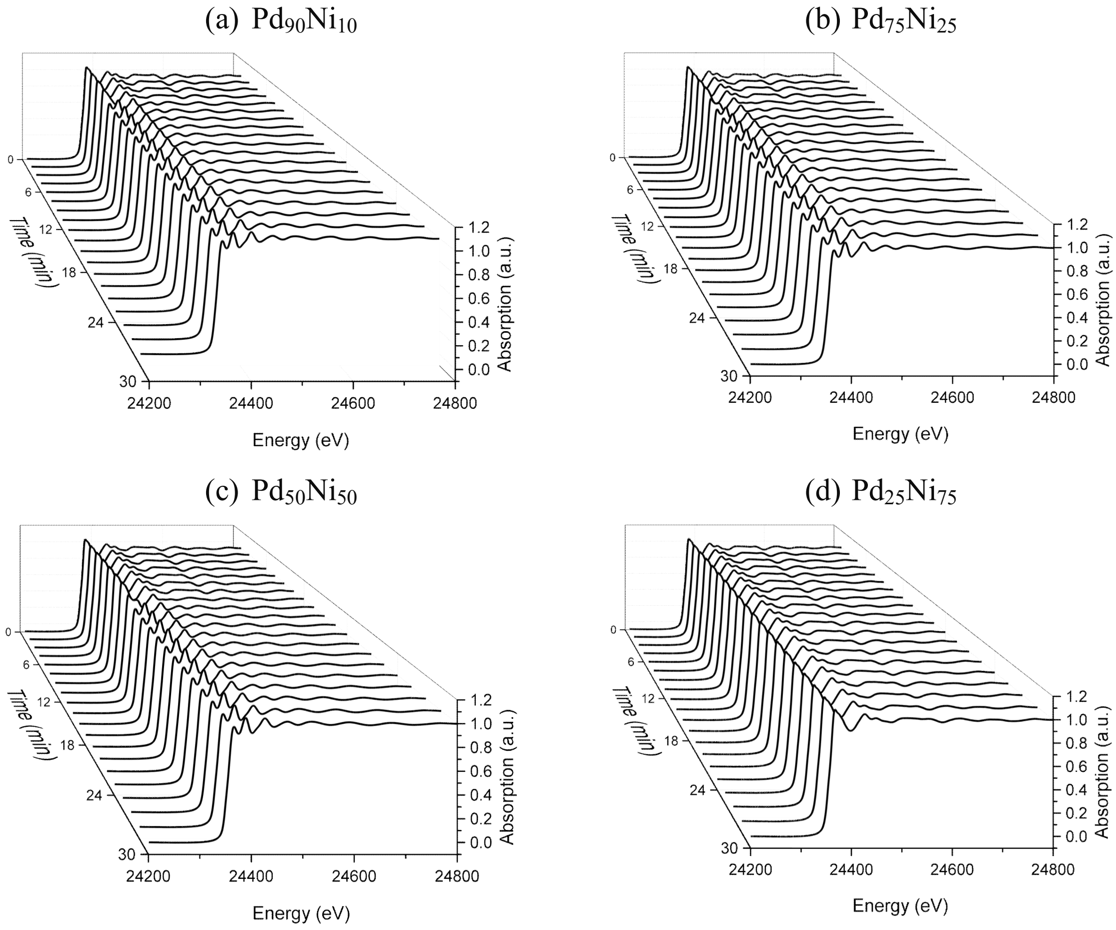

Fig. S5: Time-resolved XANES spectra at Pd K edge during exposure to 30 mL/min 4% H_2 + 96% He at RT under atmospheric pressure.

(a) $Pd_{90}Ni_{10}$

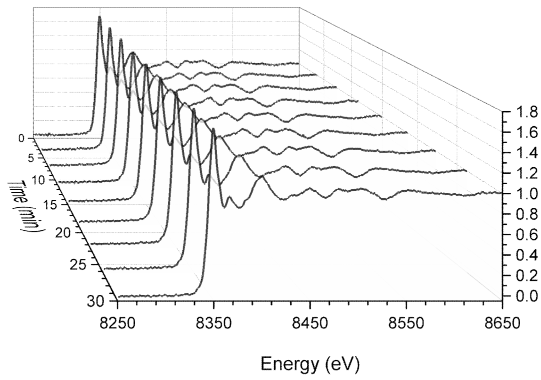

(b) $Pd_{75}Ni_{25}$

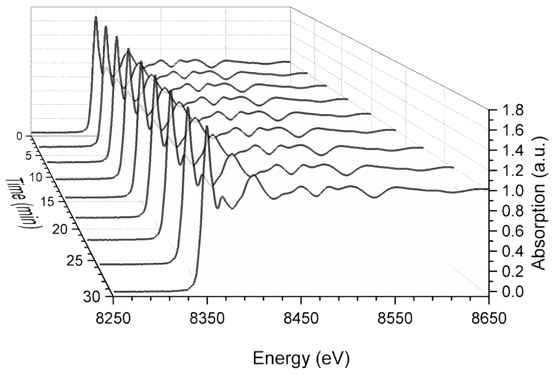

(c) $Pd_{50}Ni_{50}$

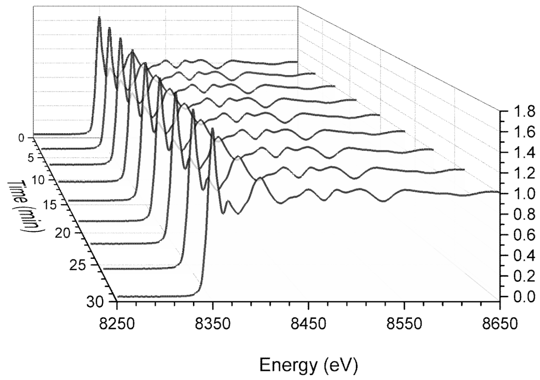

(d) $Pd_{25}Ni_{75}$

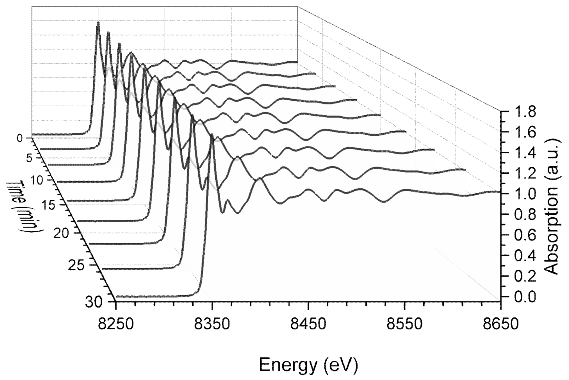

Fig. S6: Time-resolved XANES spectra at Ni K edge during exposure to 30 mL/min 4% H_2 + 96% He at RT under atmospheric pressure.

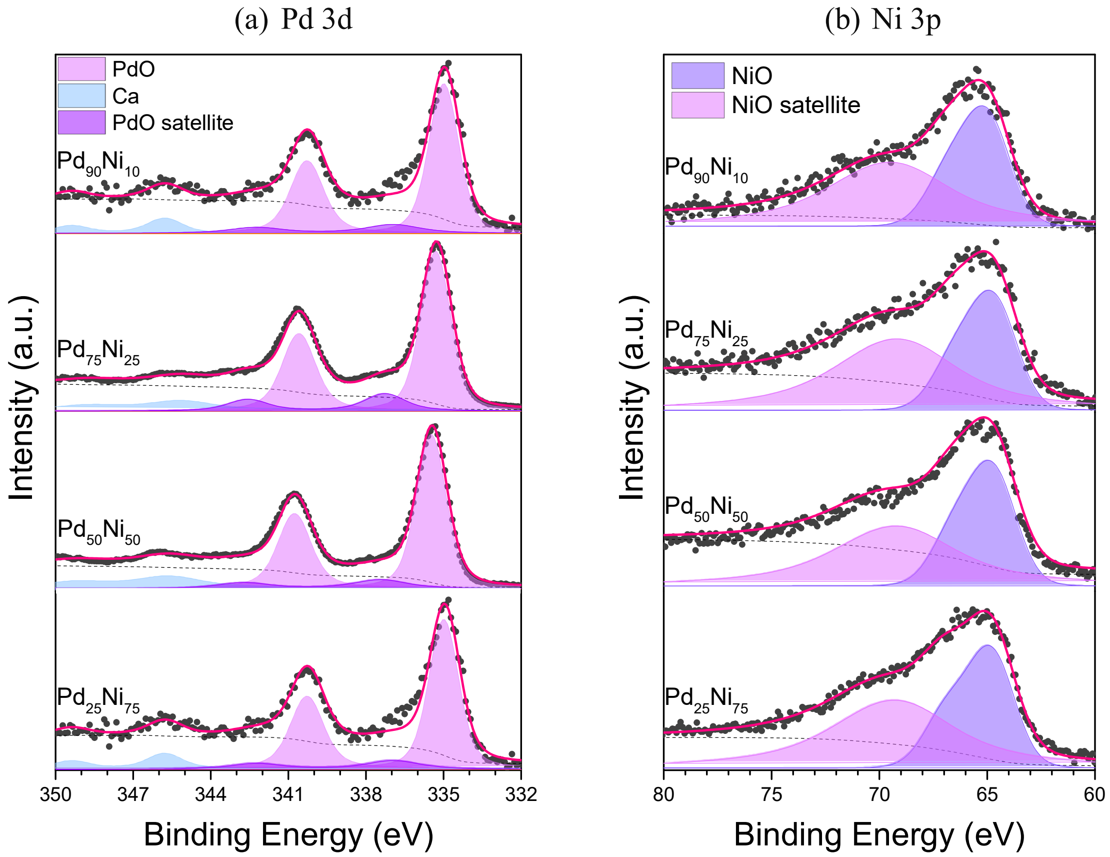

Fig. S7: XPS measurements of the as-prepared nanoparticles in the (a) Pd 3d and (b) Ni 3p region. The black dots represent the data measured, the pink line represents the fit performed and the shaded area the component as shown inset in the figure.

Note: Fig. S8 presents the XPS spectra of the Pd 3d region as a function of temperature. For the measurements, the samples were heated to 700 °C under vacuum. All the as-prepared sample, present only one Pd component related to PdO, showing that their surface is fully oxidized, as expected. During the heating treatment, it is observed the reduction of the PdO to Pd(0). For the $Pd_{90}Ni_{10}$ sample, this reduction starts before 300 °C, where two components are observed in the Pd 3d spectra. However, the full reduction of the Pd atoms does not occur before reaching 600 °C. On the other hand, for the $Pd_{25}Ni_{75}$ sample, the Pd atoms on the surface reduce almost completely before reaching 400 °C.

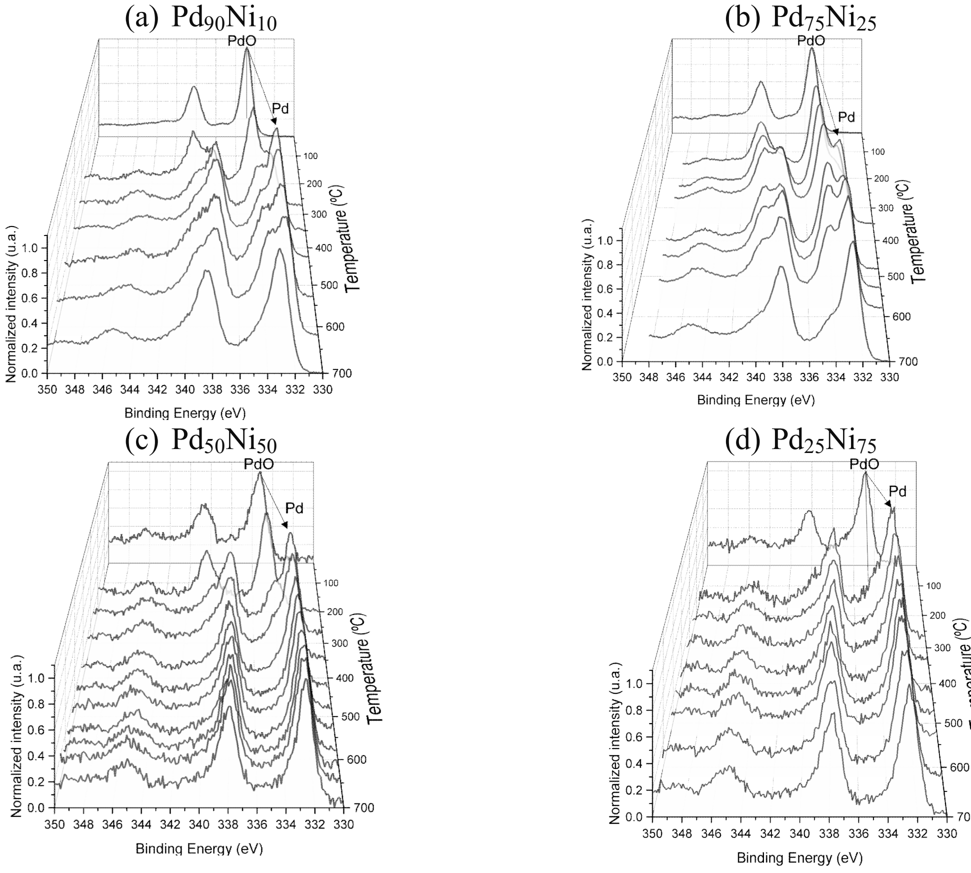

Fig. S8: XPS spectra at the Pd 3d energy region as a function of the temperature measured during the heating process under vacuum of the nanoparticles using a beam energy of 695 eV for the (a) $Pd_{90}Ni_{10}$, (b) $Pd_{75}Ni_{25}$, (c) $Pd_{50}Ni_{50}$ and, (d) $Pd_{25}Ni_{75}$ samples.

Note: Fig. S9 shows the EXAFS oscillations and the corresponding FT measured at Ni K edge for the samples under three conditions: as prepared, at 150 °C under N_2, and at RT under H_2 (all measurements done at atmospheric pressure). It is observed that all the FT are similar to the NiO standard case, presenting Ni-O and Ni-Ni scattering paths, indicating the Ni atoms are fully oxidized. Besides, no big difference is observed in the FT when the condition is changed, i.e., the as prepared, at 150 °C, and the sample under H_2 present very similar FTs. A similar effect is observed for the as-prepared and at 150 °C sample measured in the Pd K edge (Fig. S10). The as-prepared samples present Pd-O and Pd-Pd scattering peaks related to a PdO cluster, indicating the Pd atoms in the samples are fully oxidized. In addition, the position and intensity of the peaks are similar between the samples, i.e., they are independent of the amount of Pd inside the nanoparticles. In the first two steps, the samples present almost no change. However, when exposed to an H_2 atmosphere, the sample reduces, and the FT becomes similar to the Pd(0) standard.

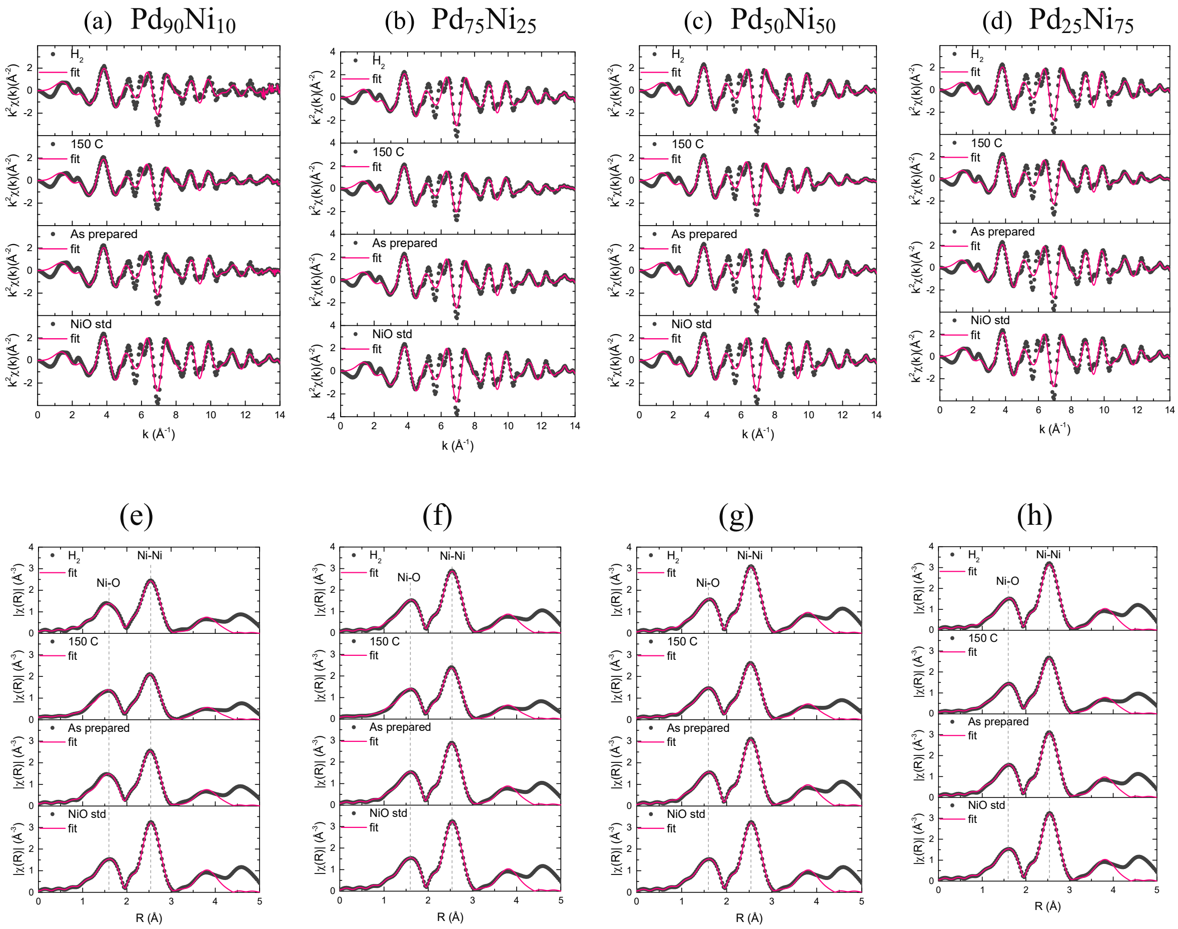

Fig. S9: EXAFS oscillations at Ni K edge and the respective FT for the (a) $Pd_{90}Ni_{10}$, (b)$Pd_{75}Ni_{25}$ (c) $Pd_{50}Ni_{50}$ and, (d) $Pd_{25}Ni_{75}$ nanoparticles. The graphics present, from bottom to up: NiO standard, as prepared, at 150 °C under 30 mL/min N_2, and at RT under 30 mL/min 4% H_2 + 96 % He (all at atmospheric pressure). The dots represent the data measured, while the pink line represents the fit performed.

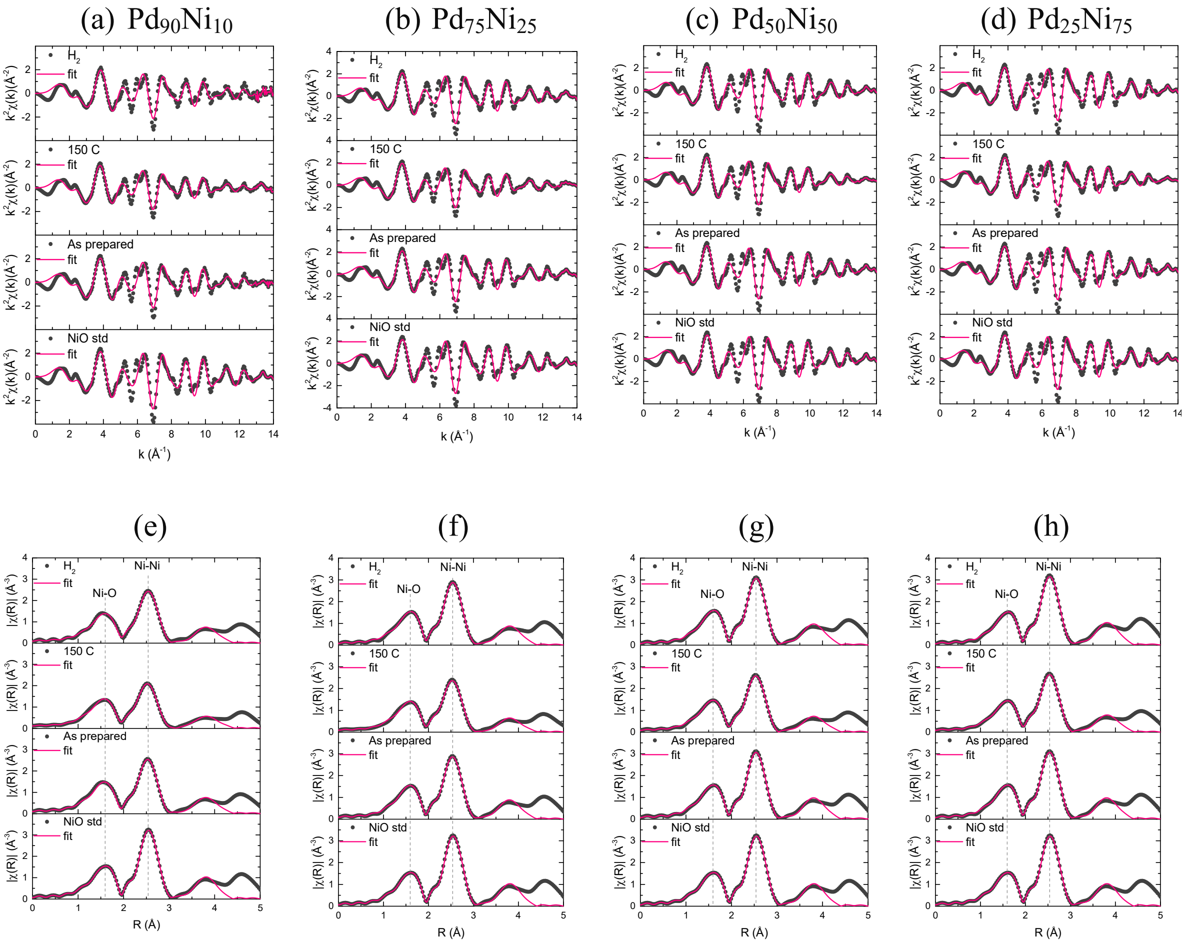

Fig. S10: EXAFS oscillations at Pd K edge and the respective FT for the (a) $Pd_{90}Ni_{10}$, (b)$Pd_{75}Ni_{25}$ (c) $Pd_{50}Ni_{50}$ and, (d) $Pd_{25}Ni_{75}$ nanoparticles. The graphics present, from bottom to up: Pd standard, as prepared, at 150 °C under 30 mL/min N_2, and at RT under 30 mL/min 4% H_2 + 96 % He (all at atmospheric pressure). The dots represent the data measured, while the pink line represents the fit performed.

Table S2: Parameters obtained from the quantitative analysis of the in-situ EXAFS data measured at the Pd K edge for the bimetallic nanoparticles during exposure to H_2 at atmospheric pressure.

**Scattering Path**		**Pd(0) standard**	**$Pd_{90}Ni_{10}$**	**$Pd_{75}Ni_{25}$**	**$Pd_{50}Ni_{50}$**	**$Pd_{25}Ni_{75}$**
Pd-Pd	N	12	11.2 ± 0.2	10.0 ± 0.2	9.4 ± 0.2	8.3 ± 0.2
	R (Å)	2.772 ± 0.001	2.818 ± 0.002	2.805 ± 0.002	2.776 ± 0.002	2.803 ± 0.001
	σ^2(Å^2)	0.0057 ± 0.0001	0.0083 ± 0.0002	0.0099 ± 0.0002	0.0090 ± 0.0002	0.0087 ± 0.0001

Table S3: Parameters obtained from the quantitative analysis of the in-situ EXAFS data measured at the Ni K edge for the bimetallic nanoparticles during exposure to H_2 at atmospheric pressure.

Scattering Path		NiO standard	$Pd_{90}Ni_{10}$	$Pd_{75}Ni_{25}$	$Pd_{50}Ni_{50}$	$Pd_{25}Ni_{75}$
Ni-O	N	6	6.000 ± 0.002	5.9 ± 0.2	5.9 ± 0.2	5.6 ± 0.2
	R (Å)	2.075 ± 0.003	2.071 ± 0.004	2.077 ± 0.003	2.077 ± 0.003	2.077 ± 0.003
	σ^2(Å^2)	0.0062 ± 0.0005	0.0081 ± 0.0006	0.0061 ± 0.0005	0.0057 ± 0.0005	0.0055 ± 0.0005
Ni-Ni	N	12	11.5 ± 0.3	12.000 ± 0.3	12.000 ± 0.008	12.000 ± 0.008
	R (Å)	2.962 ± 0.001	2.960 ± 0.002	2.962 ± 0.001	2.960 ± 0.001	2.957 ± 0.001
	σ^2(Å^2)	0.0065 ± 0.0001	0.0086 ± 0.0002	0.0073 ± 0.0002	0.0068 ± 0.0002	0.0066 ± 0.0002

Note: Fig. S11 and S12 present the AP-XPS measurements performed after annealing at 250 °C and during the exposure to 0.1 mbar H_2 at RT. In Fig. S11 and S12, as well as in Fig. 2 of the main text, the spectra were subtracted from a constant value in order to have zero as the lower value in each spectrum. In addition, the maximum value in all spectra was normalized to 1. This procedure was performed aiming to facilitate the visualization of the difference in each pair of spectra related to changes in the sample, thus removing the influence of the H_2 atmosphere in the intensity of the peaks. The difference presented in Fig. S11 and S12 (and Fig. 2, main text) was obtained by subtracting the spectrum with 0.1 mbar H_2 at RT from the annealed one under UHV. In all samples, the annealed spectra presented were measured right before the exposure to H_2. This means that the spectra used were measured after almost 2h of the sample at RT. The exception is the $Pd_{75}Ni_{25}$ sample, which was measured right when the sample achieved RT.

An observed effect in the Pd 3d energy region are small shifts in the Pd(0) component towards higher binding energies after H_2 exposure (see Fig. S11). Interestingly, a shift in the opposite direction is observed when a lower photon energy is used (see Fig. S12). The same is not observed in the Ni 3p energy region for the samples with lower Ni content, where no difference is observed between the spectra before and after H_2 exposure. However, for $Pd_{25}Ni_{75}$ nanoparticles, the Ni 3p region shifts to higher binding energies in the measurements performed with a photon energy of 695 eV (see Fig. S12 II-(d)). It is important to note that the absolute binding energy values and shifts for suspended nanoparticles are not always indicative of a chemical change. The electrostatic alignment between the particle and substrate can result in apparent shifts, as we believe is the case here as well.

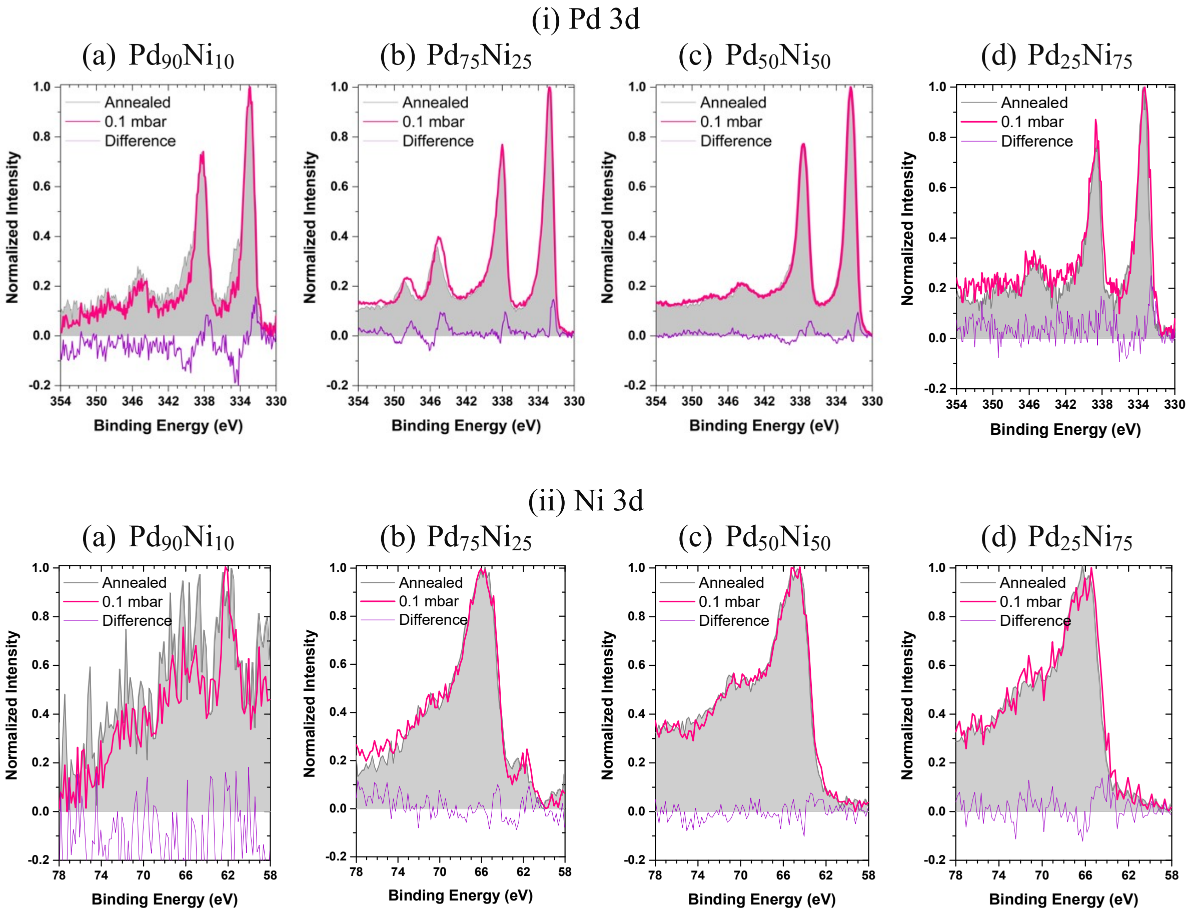

Fig. S11: AP-XPS measurements of the (a) $Pd_{90}Ni_{10}$, (b) $Pd_{75}Ni_{25}$, (c) $Pd_{50}Ni_{50}$, and (d) $Pd_{25}Ni_{75}$ in the (I) Pd 3d and (II) Ni 3p energy regions using a photon energy of 1000 eV. The solid line represents the spectrum after annealing (gray) and during the exposure to 0.1 mbar of H_2 at RT for 2h (pink). The difference between the spectrum during the H_2 exposure process and the spectrum measured after annealing is presented in purple.

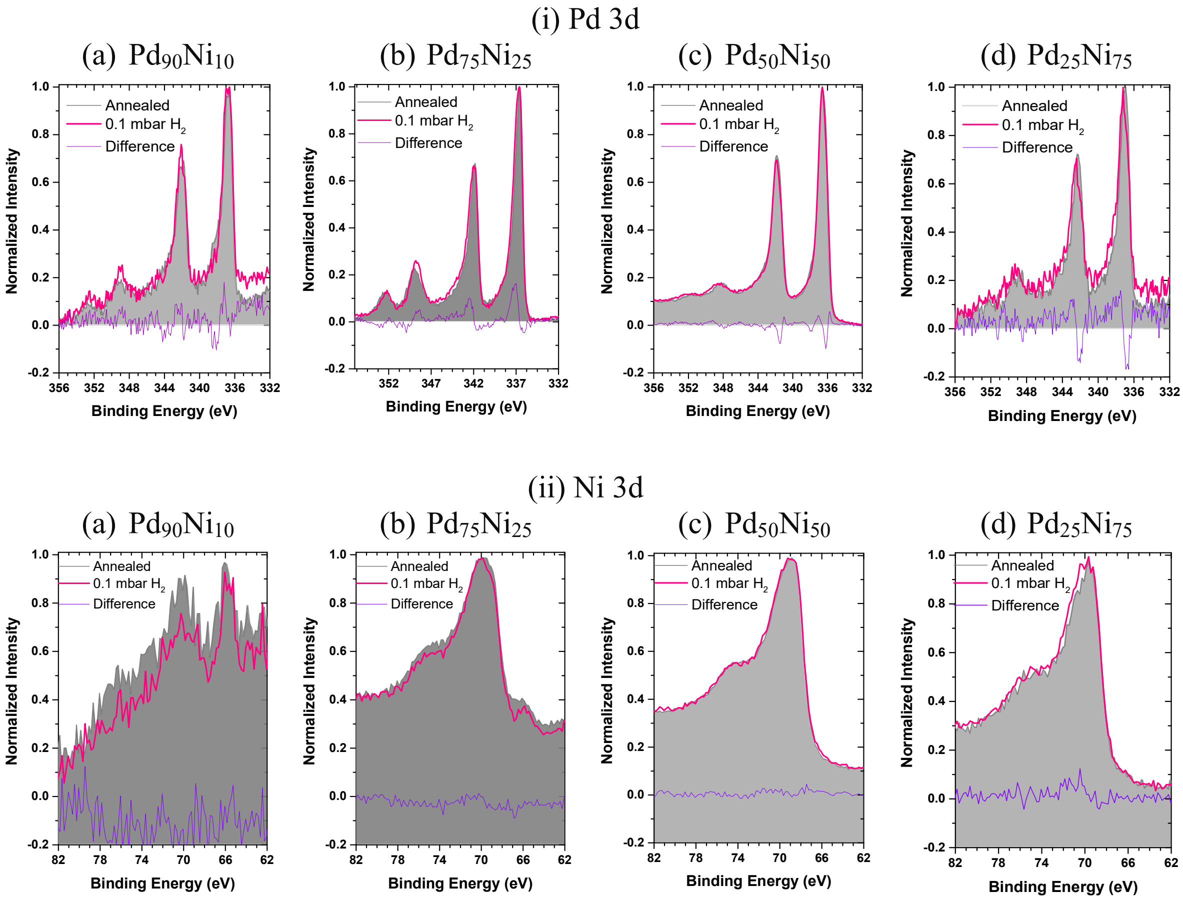

Fig. S12: AP-XPS measurements of the (a) $Pd_{90}Ni_{10}$, (b) $Pd_{75}Ni_{25}$, (c) $Pd_{50}Ni_{50}$, and (d) $Pd_{25}Ni_{75}$ in the (I) Pd 3d and (II) Ni 3p energy regions using a photon energy of 695 eV. The solid line represents the spectrum after annealing (gray) and during the exposure to 0.1 mbar of H_2 at RT for 2h (pink). The difference between the spectrum during the H_2 exposure process and the spectrum measured after annealing is presented in purple.

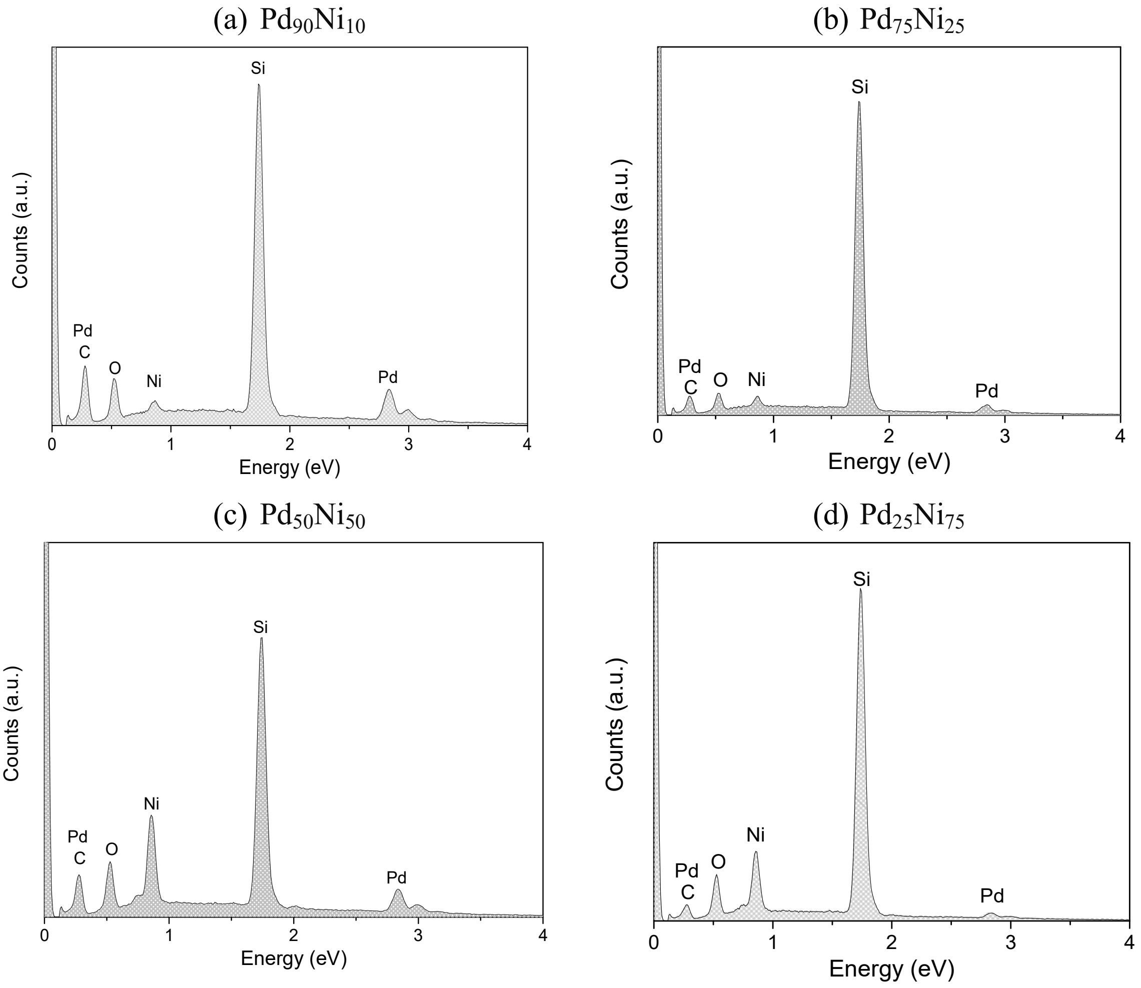

Fig. S13: EDS spectra of the as-prepared (a) $Pd_{90}Ni_{10}$, (b) $Pd_{75}Ni_{25}$, (c) $Pd_{50}Ni_{50}$ and (d) $Pd_{25}Ni_{75}$. The Si signal comes from the support used for the samples.

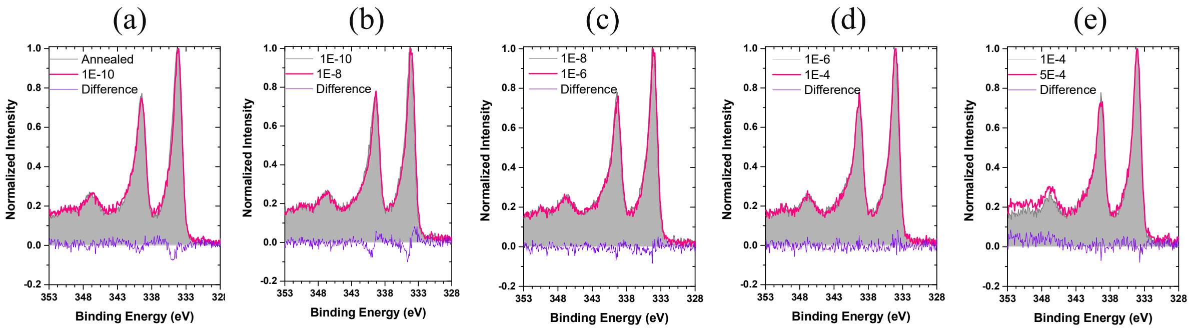

Fig. S14: Pd 3d spectra of the $Pd_{50}Ni_{50}$ nanoparticles measured by increasing the H_2 pressure to (a) 1 x 10^{-7} mbar H_2, (b) 1 x 10^{-5} mbar H_2, (c) 1 x 10^{-3} mbar H_2, (d) 1 x 10^{-1} mbar H_2 and (e) 5 x 10^{-1} mbar H_2. The pink line represents the spectra measured with a higher H_2 pressure, the gray shaded area represents the spectra measured with a lower pressure, and the purple line represents the difference between the measurements. These measurements were performed using a photon energy of 695 eV. The spectra were treated in the same way described in Fig. S10.

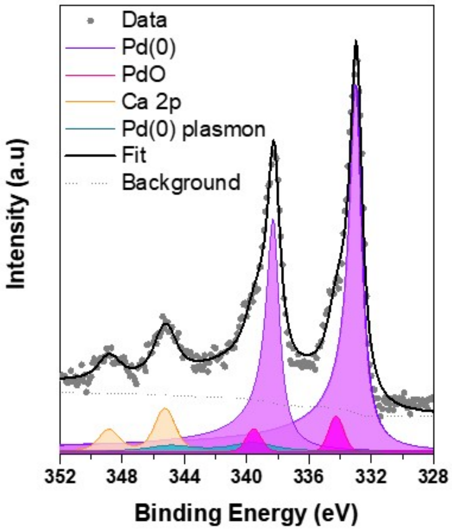

Fig. S15: Typical fitting procedure applied to the Pd 3d XPS spectra for $Pd_{90}Ni_{10}$ after the cleaning process performed under vacuum at 250 °C.

Table S4: FWHM values of the main Pd(0) component obtained from the fitting procedure of the AP-XPS spectra measured after annealing (before H_2 exposure) and during 0.1 mbar H_2 exposure for 2 h at RT using a 1000 eV or 695 eV photon energy.

	**1000 eV**		**695 eV**	
	**Annealed**	**0.1 mbar H_2**	**Annealed**	**0.1 mbar H_2**
$Pd_{90}Ni_{10}$	0.90	1.18	0.81	1.04
$Pd_{75}Ni_{25}$	0.88	0.98	0.73	0.89
$Pd_{50}Ni_{50}$	1.05	1.18	0.97	1.06
$Pd_{25}Ni_{75}$	1.02	1.34	0.90	1.16

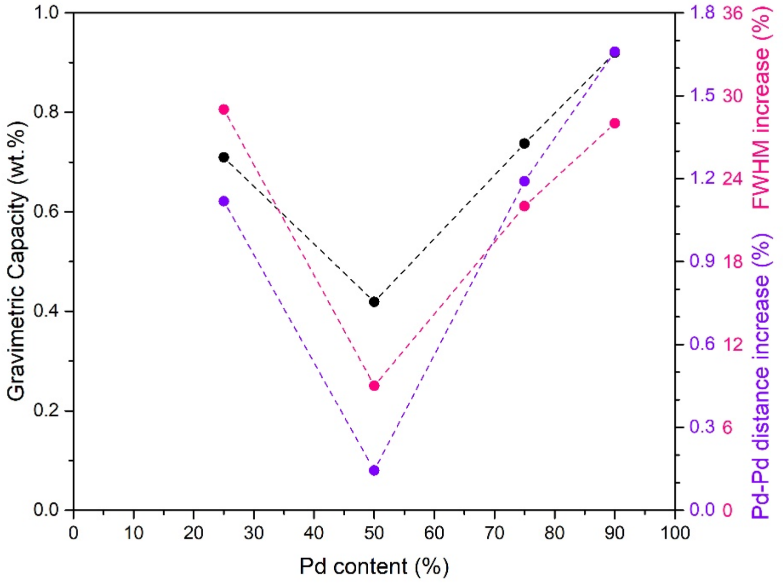

Fig. S16: Gravimetric capacity as a function of the Pd content in the nanoparticle. The Pd-Pd atomic distance (obtained from in situ XAS measurements) and FWHM of the Pd main peak (obtained from AP-XPS) is also presented as function of the Pd content in the nanoparticle.

Table S5: Gravimetric and volumetric capacity determined from the hydrogen storage measurements.

	**Gravimetric capacity (wt.%)**	**Volumetric capacity (g/L)**
$Ni_{75}Pd_{25}$	0.7	2.0
$Ni_{50}Pd_{50}$	0.4	1.2
$Ni_{25}Pd_{75}$	0.7	2.1
$Ni_{10}Pd_{90}$	0.9	2.6

Note: The AFM images show that the Pd-Ni particles with higher Pd content tend to agglomerate less than respective particles with higher content. The normal distribution gives for $Pd_{75}Ni_{25}$, mean = 26.7 nm, SD = 10.1 nm, $Pd_{50}Ni_{50}$, mean = 22.6 nm, SD = 8.5 nm, $Pd_{25}Ni_{75}$, mean = 26.3 nm, SD = 6.6 nm. These data were used for initial simulations of the particle scattering pattern using BornAgain.[1]

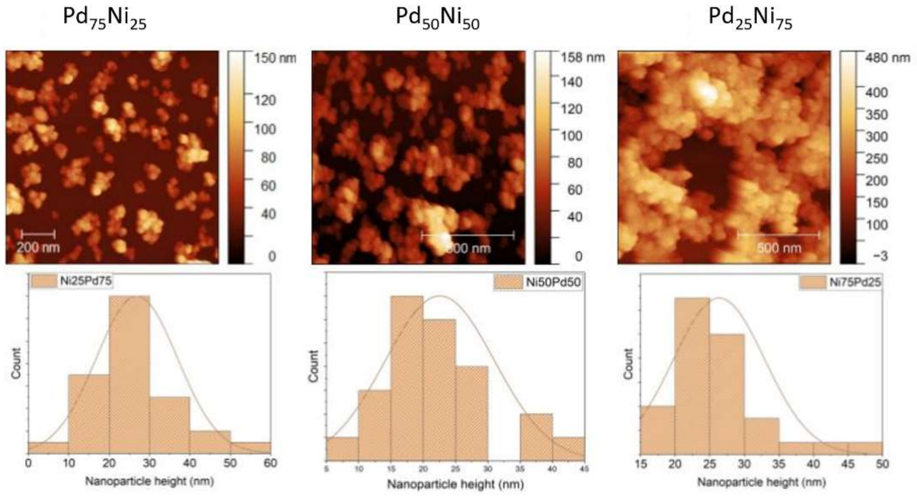

Fig. S17: AFM height measurements of the nanoparticle decorated Si wafer used to obtain the GIXS scattering curves. Distribution curves are collected for particle height only and include only particles that are directly adsorbed to the substrate.

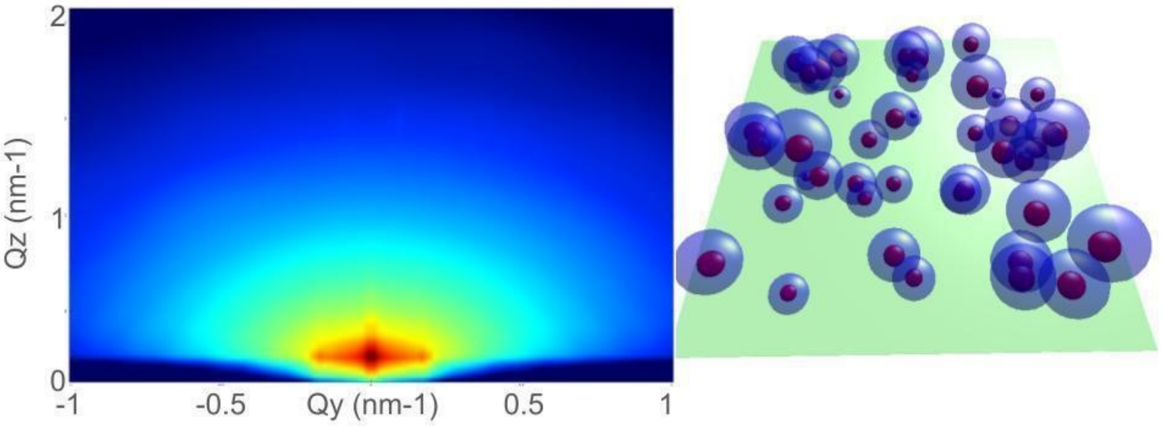

Fig. S18: Simulation of scattering spectra using BornAgain. Left simulated scattering pattern. Right used model with the substrate depicted in green the Ni shell depicted in blue and the Pd core depicted in red.

Note: To verify the validity of the obtained scattering patterns we simulated the geometry obtained by AFM utilizing the input model of BornAgain. The particle size was set to 25 nm and the standard deviation to 8.5 nm. Particle and substrate optical parameters were extracted utilizing the Optical properties tool from the Center for X-ray Optics (CXRO: https://henke.lbl.gov/optical_constants/pert_form.html) for Pd, Ni, and Si at the used photon energy of 1240 eV. Core was set to an equivalent radius of 5 nm, which corresponds to the Pd content of the $Pd_{25}Ni_{75}$ particles. All simulated geometries show the scattering pattern depicted in Fig. S18. More parameters used are depicted below:

Ni: $\delta = 0.0010246$, $\beta = 0.00047332$

Pd: $\delta = 0.0012138$, $\beta = 0.0003895$

Si: $\delta = 0.00028240119$, $\beta = 1.63896293 \times 10^{-5}$

SiO_2: $\delta = 0.000291110657$ $\beta = 3.17856538 \times 10^{-5}$

Vacuum: $\delta = 0$, $\beta = 0$

Lattice 2D:

Lattice parameter (x) = 40 nm

Lattice parameter (y) = 40 nm

Rotation angle to the lattice axis (x) = 120°

Rotation angle to the lattice axis (y) = 0°

Number of samples = 100

Decay function: 2D Cauchy with decay length of 50 nm (x) and 50 nm (y) and angular orientation of 0°

Position variance: 32 nm^2

Total Particle Surface Density = 0.000721687836487

Roughness:

Layer 1 (vacuum)

Layer 2 (SiO_2 1 nm) = 2.0, 0.8, 5 nm (root mean square, Hurst parameter, correlation lateral width)

Layer 3 (Si) = 1.0, 0.3, 5 nm (root mean square, Hurst parameter, correlation lateral width)

Beam intensity = 100000000.0

Wavelength: 1 nm

Inclination to the surface: 1.2° (vertical) and 0° (horizontal)

Spherical detector: 100 pixels, -9.2° to 9.2° (horizontal range) and 100 pixels, 0° to 18° (vertical range)

As modeling with programs such as BornAgain does not account for inhomogeneities in density within an individual form factor. Pair distance distributions (PDDF) were drawn from the out of plane line scans through the origin, to obtain information on the particle composition and shape.

The 3d electron reconstruction by GIXS may be obtained from the measurement of an object in many different angles, due to the use of many nanoparticles, this is obtained from one typical measurement.[2] We confirmed using GIXS simulations that the particles are not ordered in a defined pattern, therefore the derived form factor is not influenced by a structure factor. The out of plane cut was used to utilize the random stacking of particles sitting on other particles instead of the substrate to mimic the unoriented particle assemblies typically observed in SAXS. The electron density reconstruction was performed in the Fourier transformed GIXS curve as described in Ref 2. This electron reconstruction is not unique, since it is based on modeling. However, many tests were done with different line

cuts and similar results were obtained. In addition, besides the symmetry break of the GIXS measurements, this method initially described to SAXS is still valid.

As the total amount of electrons for each reconstruction is limited to 200 the following statements can be made for this density distributions: The higher the average density per voxel the higher the difference between the electron dense core and the shell of the nanoparticles. The higher the standard deviation the further these regions of higher density are distributed within the particle core. The electron density distributions show that the density difference between core and shell in $Pd_{75}Ni_{25}$ and $Pd_{25}Ni_{75}$ is roughly similar but way higher for $Pd_{50}Ni_{50}$.

Table S6: Bulk electron density values for Pd, PdO, Ni, and NiO.

**Bulk material**	**Electronic density**
Pd	3.06 $e^-/Å^3$
Ni	2.67 $e^-/Å^3$
PdO	2.16 $e^-/Å^3$
NiO	1.96 $e^-/Å^3$

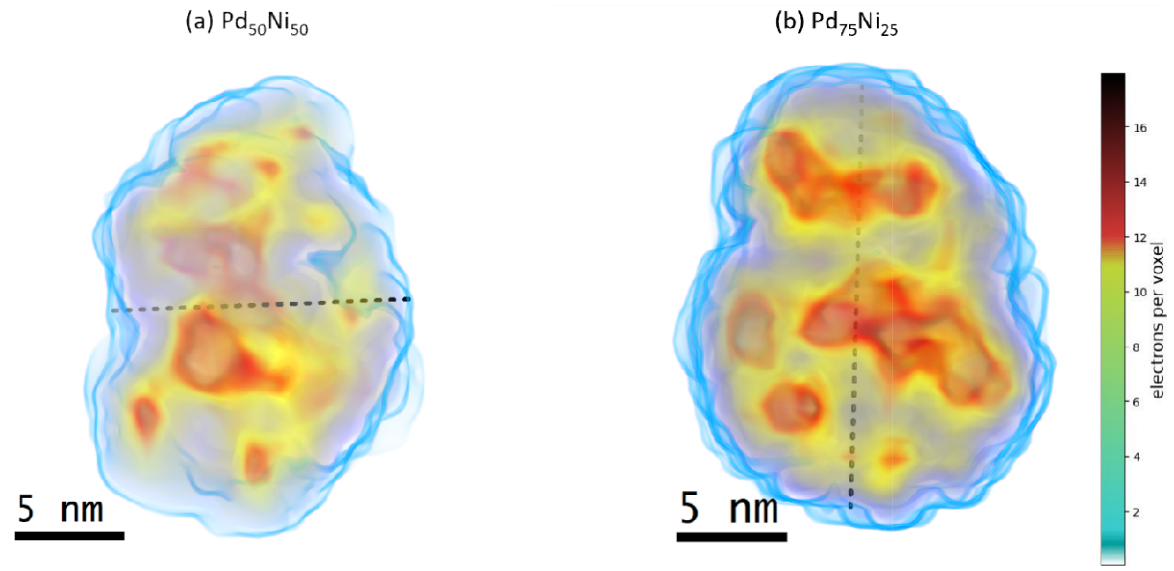

Fig. S19: Reconstruction of the electronic density after annealing for the (a) $Pd_{50}Ni_{50}$ and (b) $Pd_{75}Ni_{25}$. In red is presented the region with higher electronic density, yellow, medium electronic density, and blue, lower electronic density.

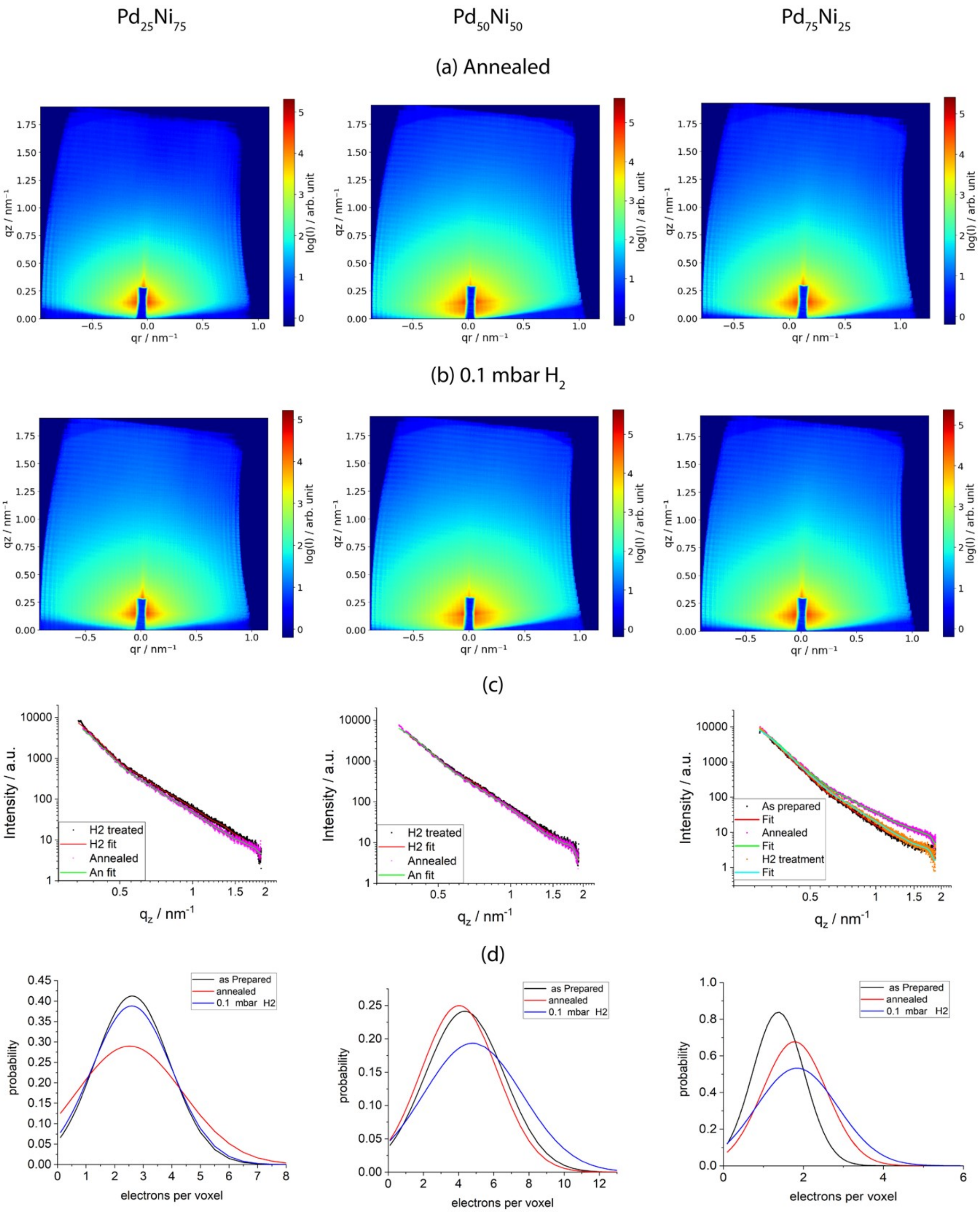

Fig. S20: (a) AP-GIXS 2D data of the samples after annealing and during the exposure to 0.1 mbar of H2. (c) Taken out of plane line cuts for the different Pd-Ni nanoparticles at all three conditions, together with the fits used to obtain the PDDFs for electron density reconstructions. (d) Distribution of electron densities within the reconstructed nanoparticles using the DENS method only voxels with electron density higher than 10% of the maximum density are considered.

(a) (b)

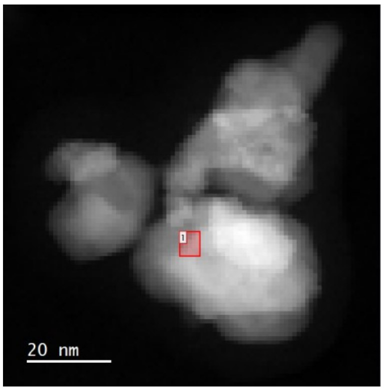

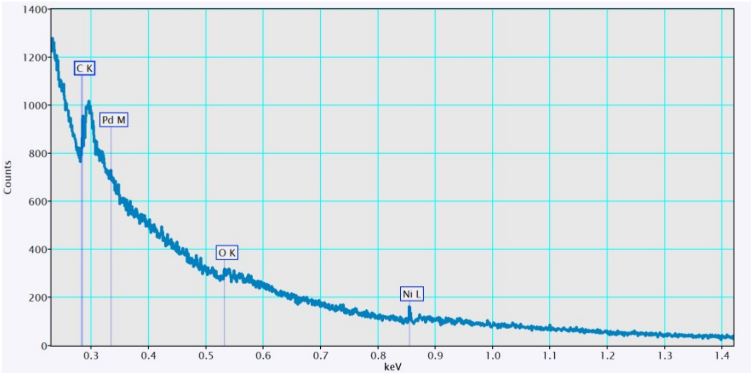

Fig. S21: (a) STEM-HAADF image of the $Pd_{25}Ni_{75}$ nanoparticles and (b) Typical EELS spectrum from the marked region in (a).

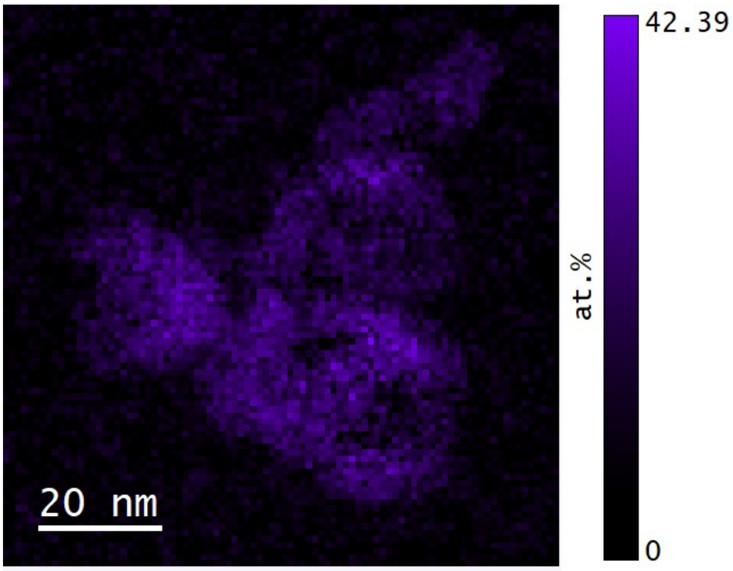

Fig. S22: Oxygen compositional map from the EELS spectrum obtained from the particle shown in Fig. 6 of main text by integrating the Oxygen K edge at 532 eV.

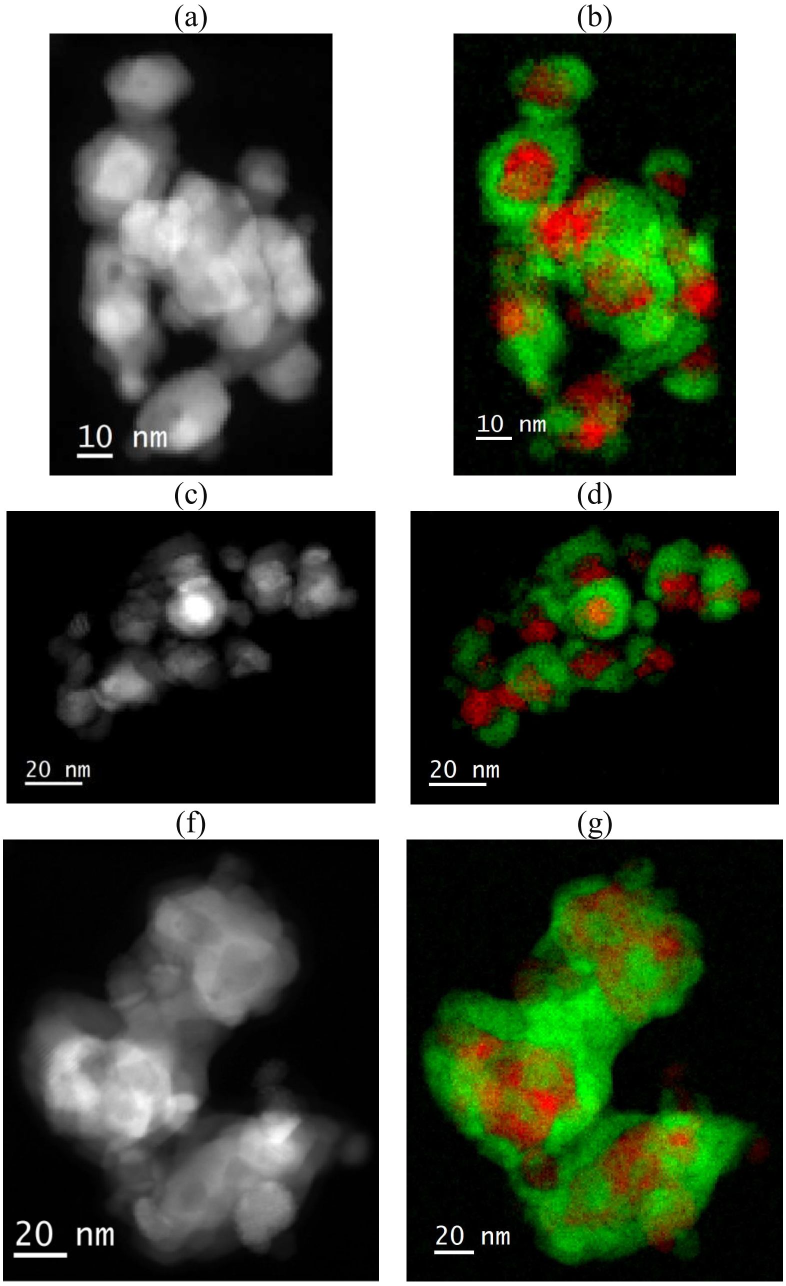

Fig. S23: Compositional mapping of as-prepared $Pd_{25}Ni_{75}$ nanoparticles obtained using STEM-EELS. On the left is presented the STEM-HAADF image while on the right side a composite map of Ni (green) and Pd(red) obtained from the Ni and Pd composition maps is presented.

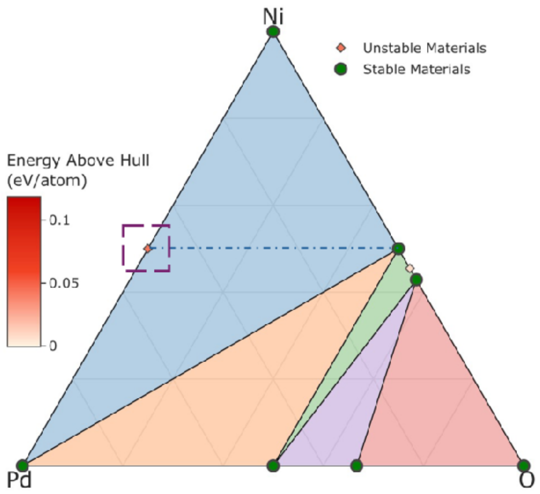

Fig. S24: Pd-Ni-O ternary convex hull obtained from the materials project. These convex hulls are calculated using the r2SCAN functional. The stable phases are shown as green dots and unstable phases are marked as diamonds. The tie lines connecting the stable phases are marked as black lines. The color of the diamonds indicates the energy above the hull. The phases $Pd_{50}Ni_{50}$ are marked in blue dashed box. The $Pd_{50}Ni_{50}$ ~ 43 meV/atom above the convex hull, respectively. Upon possible stability of these phases due to entropic effects at higher temperatures would give rise to additional tie line with the NiO phase which are marked as red dashed line.

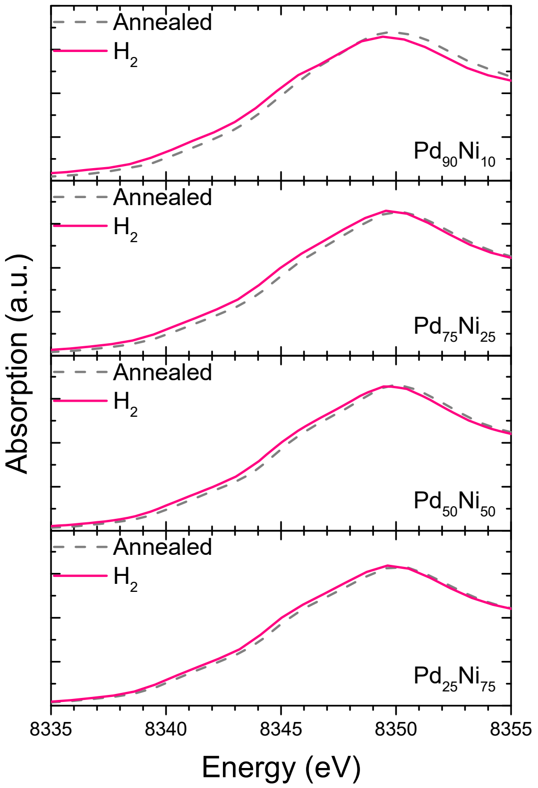

Fig. S25: XANES measurements in the Ni K edge of the samples before and during H_2 exposure.

References

(1) Pospelov, G.; Van Herck, W.; Burle, J.; Carmona Loaiza, J. M.; Durniak, C.; Fisher, J. M.; Ganeva, M.; Yurov, D.; Wuttke, J. BornAgain: Software for Simulating and Fitting Grazing-Incidence Small-Angle Scattering. *J. Appl. Crystallogr.* **2020**, *53*, 262–276.

(2) Yefanov, O. M.; Vartanyants, I. A. Three Dimensional Reconstruction of Nanoislands from Grazing-Incidence Small-Angle X-Ray Scattering. *Eur. Phys. J. Spec.* Top. **2009**, *167*, 81–86.
